# Neuroimaging of brain connectivity related to reading outcomes in children born preterm: A critical narrative review

**DOI:** 10.3389/fped.2023.1083364

**Published:** 2023-03-01

**Authors:** Kaitlyn J. Kelly, John S. Hutton, Nehal A. Parikh, Maria E. Barnes-Davis

**Affiliations:** ^1^ Division of Neonatology, Cincinnati Children’s Hospital Medical Center, Cincinnati, OH, United States; ^2^ Department of Pediatrics, University of Cincinnati College of Medicine, Cincinnati, OH, United States; ^3^ Division of General & Community Pediatrics, Cincinnati Children’s Hospital Medical Center, Cincinnati, OH, United States

**Keywords:** premature, language, reading, brain, connectome, neuroimaging, diffusion MRI, functional MRI

## Abstract

Premature children are at high risk for delays in language and reading, which can lead to poor school achievement. Neuroimaging studies have assessed structural and functional connectivity by diffusion MRI, functional MRI, and magnetoencephalography, in order to better define the “reading network” in children born preterm. Findings point to differences in structural and functional connectivity compared to children born at term. It is not entirely clear whether this discrepancy is due to delayed development or alternative mechanisms for reading, which may have developed to compensate for brain injury in the perinatal period. This narrative review critically appraises the existing literature evaluating the neural basis of reading in preterm children, summarizes the current findings, and suggests future directions in the field.

## 1. Introduction

### 1.1. Preterm birth and neurodevelopment

Premature infants are known to be at risk for brain injury, including periventricular leukomalacia, intraventricular hemorrhage, diffuse white matter injury, and cortical gray matter abnormalities (1–4). Insults related to premature delivery, such as hypoxia, ischemia, inflammation, undernutrition, and sepsis, may result in dysregulation and toxicity from microglia, injury to the oligodendrocyte precursors, and/or direct injury to axons leading to white matter dysmaturation (5, 6). Additionally, premature infants, especially those delivered at youngest gestational ages, are at risk of developmental delay, including cognitive, motor, behavioral, language, and learning deficits (1, 7–9).

Children born prematurely are less likely to be ready for school and more likely to experience educational delay, with risks increasing as birth gestational age (GA) decreases (10–12). Preterm (PT) children continue to perform below their term-born peers in reading, spelling, mathematics, and measures of executive function and behavior (8, 13–18), in one meta-analysis performing a half standard deviation below term peers in reading (13). Poor academic achievement can lead to grade failure, lower rates of higher education, lower vocational potential, and behavioral issues, which may compromise success (19, 20). Studies report two to three times higher risk of learning disability for very preterm (VPT, <32 weeks GA) and very low birth weight (VLBW, <1,500 g) children and three to five times higher risk for extremely preterm (EPT, <28 weeks GA) and extremely low birthweight (ELBW, <1,000 g) children, with many requiring remedial assistance in school (21–24). Rates of specific learning disability vary across cohorts, which may be at least partially attributed to varied definitions of learning disability, but are higher than expected in ELBW and VLBW (16, 18, 25). Though data are mixed, some cohorts demonstrate an increased rate of specific reading disability compared to term-born comparison children (TC) and rates of combined reading and mathematics disability and comorbid learning disability with intellectual disability are higher in PT children (16, 18, 25).

### 1.2. Development of the reading network and impact of prematurity

Even outside of a formal diagnosis of learning disability, premature children demonstrate deficits in reading skills, including phonological awareness, decoding, vocabulary, rapid naming, and comprehension, and are subsequently at risk for low achievement in reading (17, 18, 26–29). Emergent literacy comprises the period before formal instruction in reading when children acquire these fundamental skills for reading (30). Delays in any of the foundational components of emergent literacy can lead to later delays in reading acquisition. As language is one such crucial foundational skill for the development of literacy, it is unsurprising that language delays at younger ages are predictive of reading ability in PT children at school age (31–33). Unfortunately, deficits in reading skills do not appear to improve with time in PT, with gaps in decoding remaining stable over age at assessment and gaps in reading comprehension widening with age at assessment (26). Therefore, identification of modifiable factors that could confer resiliency in preterm children is of the utmost importance.

The development of reading is an advanced skill that harnesses the pre-existing language network, pairing it with regions of the brain involved in the recognition of visual symbols such as letters and words (orthographic processing), decoding words (phonological processing), and areas involved in semantic comprehension and attention (34, 35). Though the exact timing is fluid, reading acquisition occurs roughly in 3 stages—emergent literacy from age 3 to around 6 years, early literacy when formal instruction in reading begins (grade 1–3, roughly age 6–8), and conventional literacy or reading maintenance (grade 4, roughly age 9–10 and beyond) (34). Initial reading focuses on the decoding of words into phonemes, or units of sound, and letter and word recognition. As reading skill advances, this process becomes automatic and readers attain fluency, at which point the reader switches from learning to read to reading to learn (36).

The “reading network” in typically developing term children and adults has been well studied using neuroimaging. The dorsal stream of the reading network is involved in phonological processing and verbal repetition, involving two white matter tracts—the arcuate fasciculus (AF) and superior longitudinal fasciculus (SLF)—which connect the regions of superior temporal gyrus, angular gyrus, and supramarginal gyrus to the area surrounding the inferior frontal gyrus (IFG, includes Broca's area) (37). In term children, the bilateral dorsal tracts are initially associated with reading but quickly left lateralize, with connectivity of the left dorsal tracts positively associated with reading skill until age 10 when the association between FA of the left-sided dorsal tracts and reading skill disappears (38). The ventral stream is involved in more rapid semantic processing and orthographic recognition of words, characterized by several white matter tracts—the inferior fronto-occipital fasciculus (IFOF), inferior longitudinal fasciculus (ILF), and uncinate fasciculus (UF)—which course through the left occipitotemporal sulcus and fusiform gyrus (37, 39). These tracts connect the language network, comprised of dorsal and ventral streams (35), to the visual word form area and regions related to executive function and attention, thus comprising the reading network (39). In term children, FA of the bilateral tracts is positively associated with reading skills from age 6–10, after which the ventral tracts associated with reading begin to left lateralize as well. Ultimately, by age 10, decreased structural connectivity of the UF is associated with better reading performance (38). Some studies include as part of the reading connectome the corticospinal tracts, corpus callosum, forceps major and minor, and the cerebellar peduncles which have been associated with reading in various studies in TC (40–43).

Relatively few neuroimaging studies have investigated the development of language and reading in PT children, who are at risk for delays in both areas. Brain connectivity related to language in PT children has been more extensively studied, with some studies suggesting that preterm children may employ different structural and functional networks for language compared to TC (44–52). It is possible that preterm children who are at risk for brain injury may develop compensatory pathways for language and reading that differ from their term peers. Assessment of structural connectivity of the reading and language networks has been performed with diffusion imaging, primarily diffusion tensor imaging (DTI). Functional connectivity is being explored to assess reading, with limited studies performed with functional MRI (fMRI) and magnetoencephalography (MEG), which can be used to determine activation in task-associated cortex.

### 1.3. Imaging methods used to assess reading networks

While an extensive review of each of the imaging methodologies used to assess reading networks in PT and TC is not possible within this paper, we will briefly summarize the various techniques used in the articles discussed. Most studies evaluating reading-related skills in term and preterm children with neuroimaging use diffusion MRI (dMRI), often specifically employing diffusion tensor imaging (DTI) to assess structural connectivity. DTI is a measure of the direction of water diffusion and results have been interpreted as reflecting white matter integrity (53). Fractional anisotropy (FA) is a commonly used measure which represents the degree to which diffusion is in one direction (anisotropic), as would be expected within an axon or otherwise highly myelinated region. Other metrics include axial diffusivity, the degree of diffusion in the principal direction; radial diffusivity, the degree of diffusion in the direction perpendicular to the principal direction; and mean diffusivity, the net degree of diffusion. It is acknowledged that DTI is an oversimplified model, which is problematic if one desires a measure of “white matter integrity,” as DTI is unable to sufficiently address the problem of crossing fibers which impact approximately 90% of the voxels in the brain (54). Therefore, higher-order “tensor free” models might be preferred. One example of advanced diffusion imaging that has been used in studies of reading is Neurite Orientation Dispersion and Density Imaging (NODDI), which distinguishes between the intracellular, extracellular, and cerebrospinal fluid diffusion compartments (55). NODDI is thought to account for density of axons and increased dispersion that may contribute to unreliable fractional anisotropy results, thereby providing a more reliable picture of white matter microstructure (55). The metric of neurite density is correlated with the intensity of myelin stain and is weakly positively correlated with FA; neurite orientation dispersion assesses the tract direction of axons and provides an improved assessment of crossing fibers and connectivity (55). Another method, myelin water fraction imaging, has been proposed to be a more accurate measure of myelin histology than DTI (56). Relaxometry, a measure of relaxation time in T1 or T2 weighted MRI, might be more reflective of myelin water fraction than DTI (57, 58). Relaxometry and myelin water fraction imaging have not been widely used to study reading in preterm children, though they have been explored in term children (59).

Functional imaging allows evaluation of regions of cortex that activate during a specific task, which—theoretically—may provide a clearer picture of the network of regions involved in reading. fMRI can be performed during resting state or during an activity, during which a blood oxygen level dependent (BOLD) signal is created when neuronal activation and oxygen consumption leads to increased local blood flow causing a change in magnetization of hemoglobin molecules in the red blood cells as they shift from deoxygenated back to oxygenated (60). fMRI studies can evaluate areas of the brain used during a task in real-time, providing insight into neural connectivity by investigating areas of cortex that activate in response to specific tasks. For task-based fMRI studies, statistical contrasts can be generated to identify task (i.e., reading) associated BOLD activation vs. rest or vs. a control condition. For resting-state and for task-based fMRI studies, the time series of this BOLD activation can be correlated with the time series of other brain regions or areas of task-associated activation to give a measure of functional connectivity. fMRI has excellent spatial resolution, allowing data-driven identification of possible regions of importance during certain cognitive tasks. One of the downsides of fMRI, however, is that the temporal resolution is relatively slow compared to other modalities. MEG, which can also be used to assess functional connectivity, has sub-millisecond resolution. This fast processing is ideal for evaluating tasks which involve rapid integration of diffuse areas of the brain, such as reading and language, and has been used to assess functional connectivity related to language in preterm children (48, 50, 51). A review of all the functional connectivity metrics that can be derived from the time series data in MEG or EEG is beyond the scope of this paper. However, as noted above, a distinct advantage of these methodologies is the sub-millisecond temporal resolution that can be used to not only assess undirected functional connectivity (as in fMRI) but also directed measures of connectivity and information flux (61).

### 1.4. Behavioral assessments of reading

Reading fluency depends on several prerequisite skills, including verbal comprehension and vocabulary, phonological awareness or the ability to decode words, and rapid orthographic recognition of letters and eventually sight words (36, 62). Studies assessing reading skills often assess these foundational skills as well. Commonly used tests to evaluate language skills in English-speaking participants include the Peabody Picture Vocabulary Test, a measure of receptive vocabulary (63), and the Comprehensive Evaluation of Language Fundamentals, which assesses measures of receptive language, expressive language, and language vocabulary (64). The Comprehensive Test of Phonological Processing (CTOPP) is particularly important as it assesses decoding skills related to phonological processing and speed of retrieval through several subtests (65). Phonological processing is acknowledged to be a critical foundational skill related to future reading ability in TC and PT (32, 66). Measures that specifically assess reading skill include Gray Oral Reading Test which produces an Oral Reading Index comprised of 4 subtests assessing rate, accuracy, fluency, and comprehension (67) and the partner Gray's Silent Reading Test which assess silent reading ability; Woodcock-Johnson Tests of Achievement Basic Reading Composite, which measures decoding ability *via* the word identification and word attack subtests, and the passage comprehension subtest which assesses reading comprehension (68); Woodcock Reading Mastery Test which assesses decoding, rapid naming, passage comprehension, and fluency (69); Test of Word Reading Efficiency measures efficiency of sight word reading and decoding skills (70); Peabody Individual Achievement Test which assesses reading recognition and comprehension in additional to other academic skills (71); and Wide Range Achievement Test which assesses reading skills and comprehension in addition to spelling and mathematics (72). Most studies in term children that assess reading focus on the skills that compose reading, such as decoding or phonological awareness and rapid naming, as opposed to fluent reading (34). This is true in the literature of preterm children as well.

### 1.5. Objectives

The aim of this paper is to systematically search for and critically review the existing literature exploring structural and functional brain connectivity related to reading in preterm children. We will summarize what is known to date, review potential controversies in the field, and assess areas for further study. We will focus on the neural underpinnings of reading in preterm children and discuss whether alternative connectivity or mechanisms for reading are present in children born PT vs. TC. Investigation of these mechanisms may reveal compensatory pathways which may serve as markers of resiliency or positive adaptability, allowing PT children to overcome the risks of prematurity to achieve normal cognitive outcomes. An improved understanding of this area may ultimately lead to interventions that could help optimize outcomes and improve quality of life for premature children.

## 2. Materials and methods

**Figure 1:**
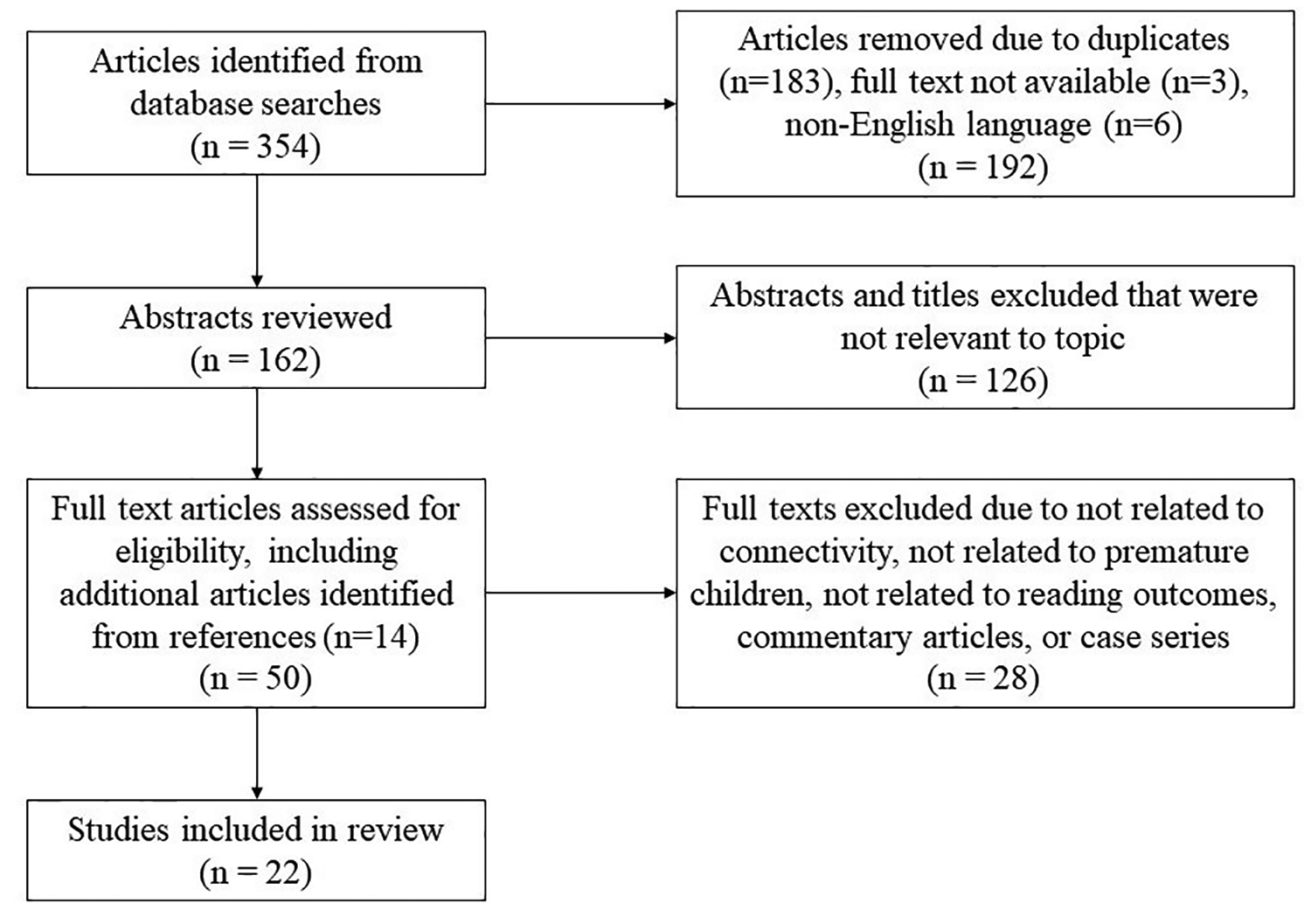
Article selection process. Flowchart describing the article selection process including initial database search with removal of duplicates, abstracts reviewed and excluded, and finally full texts reviewed and either excluded or included in the literature review.

For this review, a systematic search was performed using combination of the terms “premature” or “preterm;” “neuroimaging” or “MRI” or “connectivity” or “EEG;” and “reading” or “literacy” in the PubMed and Embase databases, yielding 167 and 123 results respectively. An additional search was performed using MeSH terms “infant, premature” or “infant, low birth weight,” and “functional neuroimaging” or “diffusion MRI” and “reading,” as a MeSH major topic and limiting results to those involving human subjects and written in the English language, yielding 64 articles. There was no restriction based on date of publication, with the search updated until October 27, 2022. A total of 354 abstracts from all searches were identified, which was reduced to 162 after removal of repeated articles and those with full text either not available (3) or not available in English (6, see Figure 1). These abstracts were screened for relevance to the topic and in total, 50 full text articles were reviewed in detail to assess for eligibility including 14 identified from references of the first search. Articles were then further excluded if not related to premature children, reading outcome, or structural or functional connectivity.

Three articles were excluded due to being commentary articles only. Three studies were identified that related reading in preterm children to findings on structural MRI, including volumetric analyses (73, 74) and degree of temporal lobe gyrification (75). These were not included due to the focus of the current study on connectivity-related imaging and reading. We identified only one EEG study relating early postnatal EEG background activity to literacy precursor skills at age 5 (76). This study was excluded due to a lack of focus on connectivity. Four case series discussing structural connectivity and reading outcome were identified from children excluded from the larger cohorts of the studies reported; these were not included but are mentioned briefly in the discussion.

Ultimately, 22 articles evaluating neuroimaging of structural or functional connectivity related to reading outcomes in preterm children were included in this review. All studies were cohort studies; no randomized control trials, systemic reviews, or meta-analyses were identified. Fifteen articles related to structural connectivity were selected that compared outcomes in preterm children to controls, assessed connectivity through diffusion imaging, and attempted to correlate connectivity to behavioral outcomes related to reading. The search revealed very few fMRI or functional connectivity studies of reading in prematurity. Four functional imaging studies (3 fMRI, 1 MEG) focused on connectivity and reading-related outcomes. An additional 3 task-based fMRI studies were included in this review due to identification of alternate areas of activation during reading tasks and an attempt to correlate these “reading networks” to reading-related metrics. It should be noted that these three articles do not use overt connectivity metrics in their studies, which is a weakness. However, they were reviewed given the dearth of available literature in this area and the importance in establishing regions of interest or “nodes” before conducting connectivity analyses. Across imaging types, articles which attempted to relate neuroimaging findings to reading outcomes and did not reveal significant associations were included. The articles were evaluated with a focus on the population studied, imaging methods employed, and results, which were compared between studies.

## 3. Results

The results of our systematic search of the neuroimaging of brain connectivity supporting reading in preterm children are reviewed below according to three neuroimaging methodologies identified: 15 dMRI, 6 fMRI, and 1 MEG study.

### 3.1. Diffusion MRI

Most of the literature of neuroimaging related to connectivity and reading-related outcomes in formerly preterm children use dMRI, typically DTI. Table 1 lists all the studies reviewed related to structural connectivity and reading-related outcomes. For clarity, the studies assessed are divided by whether the subjects assessed are at the stage of early literacy or conventional literacy, with DTI studies discussed first followed by studies using advanced dMRI methods.

**Table 1:** Characteristics of cohort populations in structural connectivity studies.

Author	Group/Trial	Imaging modality	Neuroimaging analysis	Neuroimaging metrics	TC # Age (yrs)	PT # Age (yrs)	GA of PT group (wks)	BW of PT group (g)	Severe brain injury	Behavioral assessments
Frye, 2010	Houston, TX longitudinal cohort	DTI 3.0 T scanner Diffusion protocol: 44 contiguous 3 mm sections, 21 diffusion directions (*b* = 1000 s/mm^2^)	Fiber tracts segmented using statistical parametric mapping toolbox, international consortium for brain mapping probabilistic templates ROI: SLF, superior/inferior FOF	FA, RD, pathway volume normalized by intracranial volume	*N* = 13 Age mean 16.1 (0.8)	*N* = 19 Low risk (*n* = 9), Age mean 16.0 (0.3) High risk (*n* = 10), age mean 16.2 (1.2)	<36 Low-risk: mean 31 (3.3) High-risk: mean 30 (3.0)	<1600 Low-risk: mean 1,360 ± 72.5 High-risk: mean 960 ± 466.3	Excluded if G3-4 IVH or post hemorrhagic hydrocephalus	WJ-III word identification & word attack, (decoding); CTOPP phoneme reversal (phonological awareness) and rapid naming composite (speed of retrieval); test of variables of attention
Andrews, 2010	Pittsburgh site of larger multi-site study of prematurity outcomes (Lee 2011, with Stanford)	DTI Siemens 3 T MAGNETOM Allegra Diffusion protocol: 29 axial slices of 4 mm thickness, 6 diffusion directions (*b* = 850 s/mm^2^); repeated 14 times	Preprocessed with mrVIesta, analyzed with Matlab, co-registered/aligned with SPM, oriented by AC/PC line ROI: corpus callosum segmented into genu, body, isthmus, splenium, bilateral temporoparietal region (defined by Montreal Neurological Institute coordinates)	FA (threshold ≥0.15)	*N* = 9 Age range 9–16 Mean 12 years 8 months	*N* = 19 Age range 9–16 years Age mean 11 years 11 months	Range 24–36 Mean 30.5 (325 days)	Range 572–2608 Mean 1,455 (625)	IVH—no grade designation (*n* = 3)	WJ-III word identification & word attack (decoding), passage comprehension; WASI
Feldman, 2012	Stanford site of larger multi-site study of prematurity outcomes (Lee 2011)	DTI 3 T Signa Excite 3 T1 images (averaged) Diffusion protocol: 60 slices of 2 mm thickness, 30 diffusion directions (*b* = 900 s/mm^2^); repeated 4 times, 10 non-diffusion weighted volumes	Tract Based Spatial Statistics (TBSS)—creation of white matter skeleton, laterality analysis Clusters in single tract with ≥30 voxels, continuous, FA ≥0.30 used for ROI analysis (22 ROIs in 15 major tracts)	FA (threshold ≥0.2)	*N* = 19 Age range 9–16 Age mean 13.1 (2.1)	*N* = 23 Age range 9–16 Age mean 12.5 (2)	<36 Mean 28.7 (2.5)	<2500 Mean 1,184 (431)	IVH > grade 2, echodensities or cystic lesions (*n* = 4)	WJ-III word identification & word attack, (decoding), passage comprehension; CELF-4 receptive language index, expressive language index, language memory index; PPVT-III; TROG-2 syntactic comprehension, linguistic processing speed; WASI—verbal IQ
Yeatman, 2012	Stanford site of larger multi-site study of prematurity outcomes (Lee 2011)	DTI 3 T Signa Excite Diffusion protocol: 60 slices 2 mm thickness, 30 diffusion directions (*b* = 900 s/mm^2^); repeated 4 times, 10 non-diffusion weighted volumes	Tractography *via* Automated Fiber Tract Quantification (AFQ) to create Tract Diffusion Profiles *via* 3 steps: 1. Whole brain fiber tractography *via* deterministic streamlines tracking algorithm, 2. Waypoint ROI tract segmentation, 3. Fiber tract refinement based on probabilistic fiber tracts atlas followed by fiber tract cleaning, clipping, and quantification ROI: left AF, left SLF	FA, MD, RD, AD	*N* = 18/48 Age range 9–16 48 total TC underwent scanning; 18 underwent behavioral assessments	*N* = 26 Age range 9–16	<36	<2500	IVH > grade 2, echodensities or cystic lesions (*n* = 4)	WJ-III Basic reading skills composite index (word identification & word attack)
Travis, 2015 (Human Brain Mapping)	Stanford site of larger multi-site study of prematurity outcomes (Lee 2011)	DTI 3 T Signa Excite 3 T1 scans Diffusion protocol: 60 slices, 30 diffusion directions (*b* = 900 s/mm^2^); repeated 4 times, 10 non-diffusion weighted volumes	Tractography: deterministic tractography, fiber tract segmentation and quantification *via* AFQ (Yeatman, 2012), laterality index ROI: bilateral SCP, MCP, bilateral ICP	Primary: FA (threshold ≥0.2) Secondary: AD, RD	*N* = 19 Age 9–17 Age mean 12.9 (2.2)	*N* = 26 Age 9–17 Age mean 12.8 (2.3)	<36, Range 26–34.5 Mean 28.2 (2.2)	<2500 Mean 1159.1 (427.4)	IVH > grade 2 but no cerebellar injury (*n* = 3) Ventricular dilation (*n* = 4)	WJ-III Basic reading skills cluster (word identification & word attack, assesses decoding), passage comprehension; WASI performance IQ
Travis, 2016 (Neuroimage)	Stanford site of larger multi-site study of prematurity outcomes (Lee 2011)	DTI 3 T Signa Excite scanner 3 T1 images (averaged) Diffusion protocol: 60 slices, 30 diffusion directions (*b* = 900 s/mm^2^), repeated 4 times, 10 non-diffusion weighted volumes	Tractography: deterministic tractography, fiber tract segmentation and quantification *via* AFQ (Yeatman, 2012) ROI: AF, SLF, CST, UF, ILF	Primary: FA Secondary: AD, RD	*N* = 19 Age 9–17 Age mean 12.9 (2.2)	*N* = 26 Age 9–17 Age mean 12.8 (2.3)	<36, Range 26–34.5 Mean 28.17 (2.23)	<2500 Mean 1159 (427)	IVH > grade 2, echodensities or cystic lesions (*n* = 6)	WJ-III Basic reading skills cluster (word identification & word attack, assesses decoding), passage comprehension
Dodson, 2018	Stanford longitudinal study investigating neural basis of reading in PT (enrolled 2012–2015)	DTI 3 T Discovery MR750 Scanner T1 Diffusion protocol: 70 slices, 2 mm thickness, 30 diffusion directions (*b* = 1000 s/mm^2^), 3 non-diffusion weighted volumes	Tractography: deterministic tractography, fiber tract segmentation and quantification *via* AFQ (Yeatman, 2012) ROI: left AF, left SLF, right UF	FA Secondary: AD, MD, RD	*N* = 43 Age 6 Age mean 6 years 2 months (2.3 m)	*N* = 36 Age 6 Age mean 6 years 2 months (1.8 m)	Range 22–32 Mean 29.4 (2.4)	Range 470–2180 Mean 1,325 (461)	IVH > grade 2 (*n* = 1) Small periventricular lesions (*n* = 1) Mild ventricular enlargement (*n* = 3)	WRMT word identification & word attack; CTOPP elision, sound matching, blending words; CELF-4 core language composite; WASI non-verbal IQ
Dubner, 2019	Stanford longitudinal study investigating neural basis of reading in PT (see Dodson)	DTI 3 T Discovery MR750 Scanner T1 Diffusion protocol: 70 slices, 2 mm thickness, 30 diffusion directions (*b* = 1000 s/mm^2^); 3 non-diffusion weighted volumes	Tractography: deterministic tractography, fiber tract segmentation and quantification *via* AFQ (Yeatman, 2012) ROI: corpus callosum divided into 7 segments	FA, MD	*N* = 43 Age 6 Age mean 6.2 (0.2)	*N* = 35 Age 6 Not inflamed *n* = 23 Age mean 6.2 (0.2) Inflamed *n* = 12 Age mean 6.2 (0.1)	22–32 Not inflamed: mean 30 (1) Inflamed: mean 27 (3)	<2500 g Not inflamed: mean 1,463 (293) Inflamed: mean 1,002 (552)	IVH > grade 2 (*n* = 3, all inflamed group)	WRMT-III basic skills standard scores (decoding); BRIEF global executive composite; WASI-II full scale IQ
Bruckert, 2019	Stanford longitudinal study investigating neural basis of reading (see Dodson)	DTI (age 6) 3 T Discovery MR750 Scanner T1 Diffusion protocol: 70 slices, 2 mm thickness, 30 diffusion directions (*b* = 1000 s/mm^2^); 3 non-diffusion weighted volumes	Tractography: deterministic tractography, fiber tract segmentation and quantification *via* AFQ (Yeatman, 2012) ROI: left AF, SLF, left ICP, IFOF, ILF, UF	FA	*N* = 37 Age 6 years, 8 years Age mean 6 years 2 months (2 m) and 8 years 1 month (2 m)	*N* = 34 Age 6 years, 8 years Age mean 6 years 2 months (2 m) and 8 years 2 months (2 m)	≤32 Mean 29.5 (2.4)	Mean 1,336 (468)	All IVH ≤ grade 2 Parenchymal injury (*n* = 1) Mild ventricular dilation (*n* = 2)	GORT-5 oral reading index (reading fluency & comprehension); CTOPP, CELF-4 WASI-II nonverbal IQ
Brignoni-Perez, 2022	Stanford longitudinal study investigating neural basis of reading (see Dodson)	DTI, quantitative T1 relaxometry (qR) 3 T Discovery MR750 Scanner Diffusion protocol: 70 slices 2 mm thickness, 30 diffusion direction (*b* = 1000 s/mm^2^); 3 non-diffusion weighted volumes R1 maps acquired with spoiled gradient echo (SPRF) images at 4 flip angles, 4 inversion times	Tractography: deterministic tractography, fiber tract segmentation and quantification *via* AFQ (Yeatman, 2012) ROI: left AF, SLF, ILF, UF Quantitative T1 maps calculated in mrQ (Matlab), coregistered to dMRI, R1 derived (inverse of time constant)	Primary: FA, R1 = 1/T1 (proxy for myelin content) Secondary: RD, AD	*N* = 36 *N* = 24 with usable qR data Age 8 Age mean 8.1 (0.2)	*N* = 43 *N* = 29 with usable qR data Age 8 Age mean 8.2 (0.2)	<32 Mean 29.3 (2.1)	Mean 1,310 (429)	IVH > grade 2 (*n* = 1) Small periventricular lesions (*n* = 1) Ventricular enlargement (*n* = 3)	GORT-5 Oral reading index (composite scores of fluency and comprehension)
Mullen, 2011	Yale/Brown, Follow-up component of the Multicenter Randomized Indomethacin IVH Prevention Trial	DTI Siemens Sonata 1.5 T Scanner Diffusion protocol: 40 slices, 3 mm thickness, 32 directions (*b* = 1000 s/mm^2^), 1 average	Average FA map used to manually define anatomical ROIs based on fiber bundle locations; fiber tracking from individual ROIs ROI: splenium, bilateral external capsules Voxel-based morphometry to examine volume differences	FA	*N* = 41 Age 16 Age mean 16.26 (0.34)	*N* = 44 Age 16 Age mean 16.35 (0.31)	Mean 28.3 (1.9)	Range 600–1250 Mean 994 (184)	No neonatal brain injury or IVH	TOWRE, CTOPP, PPVT-R, VMI, WISC-III
Kelly, 2016	Follow up arm of larger Victorian Infant Brain Study (VIBeS, Australia)	DTI, NODDI 3 T Siemens Magnetom Trio Diffusion protocol: (1) 25 directions (*b* = 1200 s/mm^2^), 1 *b* = 0 volume; (2) 45 directions (*b* = 3000 s/mm^2^), 6 *b* = 0 volumes NODDI fitting with *b* = 1,200 and *b* = 3000 shells	DTI *b* = 1,200 and *b* = 300 shells coregistered, normalized with *b* = 0 shell Tract-based Spatial Statistics (TBSS): alignment of FA images brought into standard space, generation of mean FA image/skeleton (FA threshold 0.2), application of NODDI	FA, neurite dispersion, neurite density	*N* = 33 Age 7 Age mean 7.6 (0.2)	*N* = 145 Age 7 Age mean 7.5 (0.2)	<30 Mean 27.5 (1.9)	<1250 Mean 974 (226)	IVH > grade 2 (*n* = 5) Cystic PVL (*n* = 5)	WRAT-4 (reading and math computation), Movement-Assessment Battery for Children-2, SDQ, WASI
Collins, 2019	Follow up arm of larger Victorian Infant Brain Study (VIBeS, Australia)	DTI, NODDI, Spherical Mean Technique (SMT) 3 T Siemens Trio System Scanner Diffusion protocol: (1) CUSP sequence 78 diffusion directions b value 400–3000 s/mm^2^), 12 *b* = 0 volumes; (2) 60 diffusion directions (*b* = 2800 s/mm^2^), 4 *b* = 0 volumes	Pre-processing in FSL, NODDI Matlab, SMT TBSS: alignment of FA images for mean FA image brought into standard space, creation of mean FA image/skeleton (threshold >0.2), nonlinear registrations applied to other diffusion images and projected onto mean FA skeleton	FA, AD, MD, RD NODDI orientation dispersion, neurite density SMT density, intrinsic diffusivity	*N* = 36 Age 13 Age mean 13.24 (0.47)	*N* = 114 Age 13 Age mean 13.22 (0.36)	<30 Mean 27.4 (1.9)	<1250 Mean 972 (239)	IVH > grade 2 (*n* = 4) Cystic PVL (*n* = 3)	WRAT-4 (reading and math computation); Kaufman Brief Intelligence Test
Thompson, 2020	Follow up arm of larger Victorian Infant Brain Study (VIBeS, Australia)	Advanced diffusion imaging with constrained spherical deconvolution modeling 3 T Siemens Scanner T1 Diffusion protocol: 45 diffusion directions (*b* = 3000 s/mm^2^), 6 *b* = 0 images	Tractography: tracts generated between ROI, connectivity estimated using AFD method (total intra-axonal volume/tract length = AFD/tract connectivity) ROI: 3 basal ganglia and thalamic, 3 cortical; defined on T1 images, warped into diffusion image space	AFD Connectivity [AFD/tract length gives a measure related to tract connectivity; AFD = measure proportional to total intra-axonal volume, summed integrals of all fiber orientation distribution (FOD) lobe within voxel traversed by tract of interest]	*N* = 19 Age 7 Mean 7.6 (0.2)	*N* = 83 Age 7 Mean 7.6 (0.3)	<30 Mean 27.6 (1.6)	<1250 Mean 1,000 (208)	IVH > grade 2 (*n* = 3) Cystic PVL (*n* = 2)	WRAT-4 word reading, math computation; Test of Everyday Attention for Children (attentional control); Working Memory Test Battery for Children; Tower of London (goal setting); Movement Assessment Battery for Children, SDQ, WASI full scale IQ
Kallankari, 2021	Finnish longitudinal cohort born 1998–2002	DTI 1.5 T GE Signa HDX Scanner T1/T2 Diffusion: 40 directions, 3 mm thickness, (*b* = 1000 s/mm^2^)	Tractography: automated probabilistic tractography (XTRACT) ROI: AF, 3 branches of SLF, ILF, IFOF, UF	FA	*N* = 21 (*n* = 20 complete data) Age range 8.8–9.3 Age mean 9.1	*N* = 56 (*n* = 48 complete data) Age 8.6–9.6 Age mean 9.0	<32 Range 24.1–31.9 Mean 28.7	Range 495–2295 Mean 1162	IVH > grade 2 (*n* = 3) PVL (*n* = 1)	YTTE (reading fluency and comprehension), Rapid naming of visual symbols, Word chain test (word reading skills)Lukilasse (spelling), Token test (verbal comprehension)

Summary of characteristics of cohorts assessed by diffusion imaging. Studies organized first by cohort or group and then by chronological order within the group. Measures used to assess reading and cognitive skills are abbreviated as follows: GORT-5, Gray's Oral Reading Test 5th edition, TOWRE, Total Word Reading Efficiency Test; WRAT-4, Wide Range Achievement Test; WRMT, Woodcock Reading Mastery Test; WJ-III, Woodcock-Johnson Test of Achievement 3rd edition. Measures used to assess language ability are abbreviated as follows: CELF-4, Clinical Evaluation of Language Fundamentals; CTOPP, Comprehensive Test of Phonological Processing; PPVT-III, Peabody Picture Vocabulary Test; TROG-2, Test for Reception of Grammar. Measures used to assess general abilities related to reading ability are abbreviated as follows: SDQ, Strengths & Difficulties Questionnaire; WASI, Weschler Abbreviated Scale of Intelligence;, WISC-III, Weschler Intelligence Scale of Children-III; BRIEF, executive function measures include the Behavior Rating Inventory of Executive Function; VMI, Development Test of Visual Motor Integration.

#### 3.1.1. Early literacy phase (6–8 years)

Most studies evaluating the white matter microstructure in younger children aged 6–8 years who have begun formal instruction in reading are performed in a cohort of VPT compared to (TC) (57, 77–79). A summary of DTI studies can be found in Table 1. These studies assess regions of interest (ROI) based on prior studies in TC of tracts related to reading, including both dorsal and ventral tracts.

Dodson compared VPT children born at 22–32 weeks and TC at age 6 with DTI and assessments evaluating language, phonological processing, and reading *via* rapid naming and decoding (77). The PT group had lower core language scores and IQ than TC but no significant difference in phonological processing scores. On DTI analysis, FA of the left arcuate fasciculus (AF) was positively associated with phonological awareness scores in both term and PT children. Conversely, FA of the right uncinate fasciculus (UF) was positively associated with language scores in the FT group only, not in PT children. Dubner evaluated DTI of the corpus callosum in the same cohort, dividing the VPT group into those with a history of prenatal inflammatory conditions (bronchopulmonary dysplasia, necrotizing enterocolitis, or sepsis) compared with those who did not and TC (78). The reading skill assessed was decoding in addition to executive function metrics. FA was significantly lower and MD higher in multiple segments of the corpus callosum in the PT group who experienced inflammatory conditions compared to the term and the preterm group without a history of a major inflammatory event. In the combined sample analysis with both TC and PT, higher FA of the occipital segment of the corpus callosum was associated with better reading and executive function, however this was not significant when separated by group. Another study from the same cohort relates DTI at age 6 with reading fluency and comprehension at age 8 in VPT and TC (79). On DTI at age 6, FA of the left AF, bilateral SLF, and left ICP positively related to reading scores at age 8 in TC. In PT, there were no significant associations between reading and FA of the left AF, right SLF, or left ICP. Significant association between reading outcome and FA of the left SLF in PT emerged only following the addition of pre-literacy skills to the model. No associations were found between reading scores and ventral stream tracts in either group.

Brignoni-Perez compared FA of ROI selected *a priori* with reading fluency and comprehension using DTI in addition to quantitative T1 relaxometry (R1), which is the inverse of the time constant in T1-weighted MRI, in 8-year-old VPT compared to TC (57). For TC, reading scores positively correlated with FA of the left AF and bilateral SLF, but no association between reading scores and FA of any tracts were seen in PT. However, on relaxometry analysis, reading scores in PT were positively correlated with R1 of the right UF, left ILF, and left SLF while no correlations were found between reading scores and R1 of any pathway in TC.

There are studies employing tensor-free analysis of dMRI from the longitudinal Victorian Infant Brain Study (VIBeS). Thompson evaluated VPT at age 7 compared to TC with constrained spherical deconvolution (CSD) modeling of cortico-striatal and thalamocortical tracts (80). Relationships between tract connectivity and reading were found only in TC, not PT, with better word reading scores weakly associated with increased tract connectivity from the left caudate and nucleus accumbens to the left lateral prefrontal cortex and the left putamen-motor tract. In a subset of the VIBeS cohort at age 7, Kelly used DTI and advanced dMRI methods (neurite density measurement with NODDI and tract-based spatial statistics, TBSS) in VPT and TC (81). In VPT only, reading scores were positively correlated with FA of diffuse fiber tracts including the cerebellar peduncles, corticospinal tract, IFOF, ILF, UF, anterior thalamic radiation, external and internal capsules, forceps major and minor, SLF, corona radiata, cingulum, fornix, posterior thalamic and optic radiation, and left superior fronto-occipital fasciculus (SFOF). However, NODDI metrics of axon dispersion and density did not specifically correlate with reading.

#### 3.1.2. Conventional literacy phase (9 years and above)

Most studies evaluating white matter tracts related to reading have been performed in cohorts of older children in later stages of reading development (conventional literacy phase) with a focus on a-priori selected tracts. A Finnish cohort study of 9-year-old VPT and TC correlated DTI with reading fluency, comprehension, rapid naming, word reading, verbal comprehension and spelling (82). In VPT, increasing FA of the left AF and bilateral SLF positively related to rapid naming scores. Rapid naming is an important factor in reading ability (82, 83). FA of left SLF (branches 2, 3) was positively associated with verbal comprehension and spelling scores. In growth restricted VLBW specifically, there was a positive association with reading comprehension scores and FA of the left IFOF, a ventral stream pathway. In TC, correlation of FA of the left SLF (branch 1) was found with rapid naming, but most tracts assessed were not associated with reading outcomes. A DTI study assessing 16-year-old adolescents stratified both by reading ability and birth group, compared TC with PT (who were further divided into “low-risk” or “high-risk” based on severity of neonatal complications) (84). The study correlated FA of the SLF, IFOF, and SFOF with decoding, phonological awareness, and rapid naming. Across groups, FA of the left SLF decreased and RD increased as reading performance increased in letter-word identification and phoneme reversal. FA of the right SLF decreased as attention performance decreased across groups. Direction of association with FA was consistent for PT and TC. Mullen related DTI in a subset of the cohort from the Multicenter IVH Prevention Trial at age 16 to sight reading, non-word decoding, and phonological awareness (85). Despite the finding of lower FA of the corpus callosum in VPT compared to TC, there was no correlation of FA of the corpus callosum with reading scores. VPT exhibited a significant positive correlation between FA of the bilateral UF and receptive language scores along with rapid naming scores.

Most studies of older PT children investigate a cohort of 9- to 17-year-olds enrolled in a multi-site study assessing cognitive outcomes of prematurity, with 4 reports from the Palo Alto arm (86–89) and 1 from the Pittsburgh arm (90). The PT group is heterogeneous in age and degree of prematurity (<36 weeks gestation). Testing of verbal IQ, receptive and expressive language, verbal memory, linguistic processing speed, syntactic comprehension, single-word reading, pseudoword reading, and reading comprehension were performed with both groups scoring within normal limits. DTI was analyzed with TBSS in a subset of this cohort (86). For PT, there were positive correlations between verbal IQ, linguistic processing speed, syntactic comprehension, and decoding with FA in 15 tracts forming a diffuse bilateral network including the corpus callosum, forceps major and minor, bilateral anterior thalamic radiation, bilateral corticospinal tracts (CST), bilateral IFOF, bilateral ILF, bilateral SLF, and bilateral UF. FA of all tracts positively correlated with syntactic comprehension and decoding. Language and reading scores positively associated with FA of the corpus callosum, forceps minor, left SLF, bilateral ILF, right anterior thalamic radiation, right corticospinal tract, and bilateral IFOF. Regression analyses identified the most important tract predictors of a given outcome: right IFOF for verbal IQ and syntactic comprehension, bilateral forceps minor for receptive vocabulary and verbal memory, right anterior thalamic radiation for linguistic processing speed, genu of the corpus callosum for decoding, and left UF for reading comprehension. There were no statistically significant associations in TC between FA of any tract and several behavioral measures. Travis performed tract segmentation in the Palo Alto cohort using predefined ROIs associated with reading, including bilateral anterior SLF, AF, CST, UF, and ILF, which were then correlated with reading decoding and comprehension (89). Positive correlations were found in PT between decoding and FA of the bilateral anterior SLF, left AF, and bilateral CST as well as between comprehension and FA of the left anterior SLF, left UF, and right CST. TC had negative correlations between decoding and FA of the left anterior SLF, bilateral CST, and bilateral UF and negative correlations between comprehension and FA of the left anterior SLF, left UF, and left AF, in contrast to the TBSS study of the same cohort by Feldman (86). The proof of concept study by Yeatman evaluating automatic fiber tract quantification (AFQ) methodology demonstrated a positive correlation in PT between single word reading skills and FA of the left AF and left SLF, while a negative correlation was found in TC between single word reading skills and FA of the left AF (87).

Two additional reports from the same multi-site study of reading and language evaluate connectivity of specific structures: the cerebellum and the corpus callosum. Decoding and reading comprehension were positively associated with FA of middle cerebellar peduncles (MCP) in both PT and TC (88). Negative associations were demonstrated in both groups between FA of the SCP and ICP and decoding and reading comprehension, though when controlled for the other skill, only FA of the left ICP remained significantly correlated with decoding and FA the right SCP with comprehension. The Pittsburgh arm of this multi-site study correlated decoding and passage comprehension with DTI of the corpus callosum (90). Lower FA of the genu, body, and splenium of the corpus callosum was found in PT compared to TC. Increasing FA of the body of the corpus callosum positively related to word identification scores in both groups.

There is one study that uses advanced diffusion imaging to evaluate connectivity related to word reading in PT. A subset of the VIBeS cohort was assessed at age 13 using tensor-free techniques (NODDI and Spherical Mean Technique) to complement their DTI analysis with TBSS, providing more reliable assessment of white matter integrity than FA alone (91). Though significant correlations were found between connectivity metrics and mathematics outcomes, analysis relating FA or NODDI of any tracts with reading outcome was not significant.

### 3.2. Functional MRI

The literature assessing functional connectivity related to reading in preterm children is limited. 3 fMRI studies reported connectivity metrics and attempted to relate connectivity with behavioral measures related to reading. These studies are listed in Table 2. There are 3 additional studies which report task-based fMRI results that suggest alternative areas of activation in reading-related tasks in preterm children. Though no connectivity metrics are reported, these studies are related to the reading network used by preterm children. These studies have been included in an effort to discuss all available studies that speak to the reading network in preterm children (Table 3).

**Table 2:** Characteristics of cohort populations in functional connectivity studies.

Author	Group/Trial	Imaging modality	fMRI/MEG paradigm	Neuroimaging metrics	TC # Age (yrs), (SD)	PT # Age (yrs), (SD)	GA of PT group (wks), (SD)	BW of PT group (g), (SD)	Severe brain injury	Behavioral assessments
Gozzo, 2009	Yale/Brown, Follow-up component of the Multicenter Randomized Indomethacin IVH Prevention Trial	fMRI GE Signa LX T1 localizing scan fMRI acquisition: BOLD contrast gradient echo, echo planar pulse sequence, 102 images per stimulus type = 306 images per experiment	Task-based fMRI subtraction paradigm involving 3 stimuli of the Ugly Duckling (Peterson 2002) to assess differential brain activity needed for semantic processing (1 vs. 2) and phonologic processing (2 vs. 3)	Functional connectivity measured as correlation between mean BOLD time course of Wernicke's area (BA22) and 11 other ROIs in primary language regions and right sided-homologues	*n* = 24 Age 7–9 Age mean 8.7 (0.6)	*N* = 54 Age range 7–9 Age mean 9.2 (0.7)	Mean 28.2 (1.9)	600–1250 Mean 977 ± 169.5)	No IVH No low pressure ventriculo-megaly	PIAT-R reading recognition, reading comprehension, mathematics, PPVT-R, WISC-III
Myers, 2010	Yale/Brown, Follow-up component of the Multicenter Randomized Indomethacin IVH Prevention Trial	fMRI	Task-based fMRI paradigm (Frost 2009): event-related cue identity task requiring match/mismatch judgment between pictures and words presented aloud, or read in printed form, responses *via* button, 8–10 runs per subject	Functional connectivity maps created with strength of connections to reference area (Wernicke's area, L BA22), regional connectivity measured to 3 ROIs (inferior L SMG, inferior R SMG, R Broca's area homologue BA 44/45)	*N* = 36 Age 16 (0.4)	*N* = 31 Age 16 (0.3)	Mean 27.7 (2.0)	Mean 933.1 (189)	No IVH No PVL No low pressure ventriculo-megaly	TOWRE, CTOPP, PPVT-R, WISC-III full scale IQ, performance IQ, verbal IQ, verbal comprehension index
Constable, 2013	Yale/Brown, Follow-up component of the Multicenter Randomized Indomethacin IVH Prevention Trial	fMRI 3 T Siemens Tim Trio scanner T1 localizing scan, anatomical scan fmRI acquisition: T2 sensitive gradient-recalled single shot echo-planar pulse sequence, runs of 190 volumes (5 min scan) discarding the first 4	Resting state fMRI: voxel-based contrast maps reflecting functional connectivity, ROI identified and used for seed-based connectivity analysis creating ROI-whole brain connectivity map	Intrinsic connectivity contrast degree (ICC-d) Seed-to-whole brain connectivity analysis (time course of reference region correlated with time courses of other voxels to create r-values map, converted to z-values, averaged to yield a strength of correlation map)	*N* = 19 Age range 18–20 Age mean 19.7 (1.1)	*N* = 19 Age range 18–20 Age mean 20.1 (0.9)	Mean 28.2 (1.8)	Range 600–1250 Mean 974.6 (163.2)	No IVH No PVL No low pressure ventriculo-megaly	CTOPP rapid naming (digit, letter), phonological awareness (blending/segmenting non-words); PPVT-R; WISC-III full-scale IQ, performance IQ, verbal IQ, verbal comprehension index
Frye, 2009	Houston, TX longitudinal cohort	MEG (WH3600, 4D Neuroimaging) with 248 axial gradiometers in cryogenic dewar, signal continuously sampled at 500 Hz, bandpass filter 0.1–50 Hz, ERFs extracted, average epochs low pass filtered at 20 Hz; T1 MRI for dipole localization (3 T Siemens Sonata)	6 min real-word and non-word rhyme tasks with 3 min break between tasks, participants use response pad to answer, 68 total trials ROI: Broca's/prefrontal areas, frontal/supplementary motor areas, middle temporal gyrus, superior temporal/Heschl's gyri, supramarginal/angular gyri	Number of dipoles (NOD, activation over a 4 ms period) in ROI over a specific time interval	*N* = 11 Age 16 Mean 16.3 (0.17)	*N* = 20 Age 16 Low risk *n* = 10 [mean age 16.3 (0.3)] High risk *n* = 10 Mean 15.6 (0.32)	<36 Low risk: mean 30.5 (1.2) wks High risk: mean 27.0 (1.7)	<1600 Low risk: mean 1,265 (96) High risk: mean 825 (208)	Excluded if G3-4 IVH or post hemorrhagic hydrocephalus	WJ-III word attack, letter-word identification; CTOPP phoneme reversal, rapid naming; Stanford-Binet 4th ed quantitative skills scores, Continuous performance task inattention

Summary of characteristics of cohorts assessed by fMRI or MEG to assess functional connectivity. Studies are organized first by cohort/group and then by chronological order within the group. Behavioral testing related to reading abilities are abbreviated as follows: PIAT-R, Peabody Individualized Achievement Test – Revised; TOWRE, Test of Word Reading Efficiency; WJ-III, Woodcock-Johnson III Tests of Achievement. Abbreviations for testing related to language/reading precursor abilities: CELF, Comprehensive Evaluation of Language Fundamentals; CTOPP, Comprehensive Test of Phonological Processing; PPVT-R, Peabody Picture Vocabulary Test – Revised. General abilities testing abbreviations are as follows: WISC-III, Weschler Intelligence Scale of Children-III.

**Table 3:** Characteristics of cohort populations in functional task-based studies related to the reading network.

Author	Group/Trial	Imaging modality	fMRI/MEG paradigm	Neuroimaging metrics	TC # Age (yrs), (SD)	PT # Age (yrs), (SD)	GA of PT group (wks), (SD)	BW of PT group (g), (SD)	Severe brain injury	Behavioral assessments
Rushe, 2004	Long term follow-up study of London cohort of adolescents born PT from 1979 to 1989	fMRI 1.5 T GE Signa System retrofitted with Advanced NMR hardware fMRI acquisition: 100 T2 weighted MR images with BOLD at each of 14 non-continuous near axial planes near intercommissural line (T*E* = 40 ms, T*R* = 3000 ms, slice thickness = 7.7 mm)	Task-based fMRI paradigm: 10 alternating phases, 6 stimulus pairs each, 30 sec/block in target task-control task format; target task = rhyme judgments about pairs of non-words; control task = letter-case judgment task (strings of consonants, decide if same pattern)	# voxels Fundamental Power Quotient (FPQ)	*N* = 6 Age mean 18.17 (1.29)	*N* = 6 Age 18.83 (1.47)	Mean 28.83 (2.64)	Mean 1,360 (237)	Ventricular enlargement (*n* = 6)	Rhyming task to assess phonological processing in fMRI Did not correlate to behavioral measures of reading in this study; prior study in the cohort assessed reading ability with Schonnel reading and spelling test to assess reading age (PT < TC) (Stewart, 1999)
Ment, 2006	Yale, Follow-up component of the Multicenter Randomized Indomethacin IVH Prevention Trial	fMRI T1 reference scan 1.5 T Siemens Sonata scanner fMRI acquisition: gradient echo, echo-planar pulse sequence, total 300 planar images per slice per run	Task-based fMRI subtraction paradigm involving 3 stimuli of the Ugly Duckling (Peterson 2002) to assess differential brain activity needed for semantic processing (1 vs. 2) and phonologic processing (2 vs. 3)	% BOLD signal change at ROIs in contrast maps for condition 1 vs. 2 (semantic) and 2 vs. 3 (phonologic)	*N* = 10 Age 12 Age mean 12.3 (0.7)	*N* = 14 Age 12 Mean 12.3 (0.5)	Mean 27.9 (1.5)	Range 600–1250 Mean 919.9 (154.9)	No IVH No PVL	TOWRE, Gray Silent Reading Test, CTOPP, CELF-3, PPVT-R, WISC-III
Van Ettinger-Veenstra, 2017	Swedish longitudinal follow up cohort of VLBW born 1998–1999	fMRI Philips Ingenia 3 T scanner T1 structural images (301 slices) fMRI acquisition: single shot gradient-echo echo planer image sequence sensitized to T2 BOLD changes (248 dynamics)	Task-based fMRI: word-pair task with 4 language choice conditions presented in block design, (1) phonological choice, (2) orthographic choice, (3) semantic judgment condition, (4) line orientation baseline condition; 5-word pairs per block; 20-word pairs per condition, 80 total word pairs presented; answer *via* button response	Degree of group activation measured in 3 contrast images of each condition compared to baseline (phonological, orthographic, semantic) 12 pre-defined ROIs (Talaraich Daemon labels atlas): bilateral IFG, MTG, STF, AG, SMG, FG	*N* = 13 Age range 12–14 Age mean 13.0 (0.2)	*N* = 13 Age range 12–14 Age mean 13.5 (0.6)	29 + 2 (15 days)	<1500 g 1,046 (347)	No IVH > Grade 2 No PVL	WISC-IV block design (visuo-spatial processing), vocabulary (verbal comprehension) Task performance measured in accuracy and reaction time

Summary of characteristics of cohorts assessed by fMRI that do not report functional connectivity metrics. Behavioral testing related to reading abilities are included and in some cases abbreviated as follows: Gray's Silent Reading Test, TOWRE, Test of Word Reading Efficiency. Abbreviations for testing related to language/reading precursor abilities: CELF, Comprehensive Evaluation of Language Fundamentals; CTOPP, Comprehensive Test of Phonological Processing; PPVT-R, Peabody Picture Vocabulary Test – Revised. General abilities testing abbreviations are as follows: WISC-III, Weschler Intelligence Scale of Children-III.

#### 3.2.1. Early literacy phase (6–8 years)

All studies identified investigating reading-related functional connectivity in PT were performed in VLBW children from the Multicenter Indomethacin IVH Prevention trial. These studies assessed reading and language skills. They are not longitudinal in nature due to use of varying subsets of participants and different tasks. Of these, 1 study involves the early literacy phase of reading attainment. Gozzo compared 7- to 9-year-old PT and TC, correlating connectivity on fMRI with reading recognition and comprehension (44). Wernicke's area (Left Brodmann Area 22) was selected as the reference ROI and connectivity was assessed to canonical left-sided language areas and their right-sided homologues. PT exhibited different patterns of connectivity compared to TC with increased cross-hemispheric activity and involvement of right sided-homologues. Specifically, increased connectivity was seen in PT from Wernicke's area to the right and left supramarginal gyri and the right IFG (homologue of Broca's area). In this study, correlations were not found between connectivity and behavioral metrics of reading. This is the only study of functional connectivity in PT in the early literacy phase at the time of this review.

#### 3.2.2. Conventional literacy phase (9 years and above)

Another study from the Multicenter IVH Prevention Trial assessed functional connectivity correlated with reading scores [Test of Word Reading Efficiency (TOWRE) assessing sight word reading and decoding], phonological awareness, and language in PT and TC at 16 years of age (47). Wernicke's area was used as a reference region and connectivity was assessed between this area and 3 ROIs that were significant in the study by Gozzo: bilateral supramarginal gyri and the right-sided homologue of Broca's area in the IFG (44). No significant correlations were found between connectivity in these pathways and reading or phonological awareness scores. However, correlations with language measures demonstrated increased strength of the alternative pathway between the left-sided Wernicke's area and the inferior portion of the right supramarginal gyrus was inversely related to receptive vocabulary. Subjects whose mothers had low levels of education also had increased connectivity strength in this pathway.

In a third study from the Multicenter IVH Prevention Trial, connectivity during resting state fMRI at 18–20 years was related to rapid naming and phonological awareness for PT and TC (92). The intrinsic connectivity contrast degree map identified an area in the left cerebellum in PT compared to TC which was used as a reference region for whole brain connectivity analysis. In PT, increased connectivity was found from this seed region in the left cerebellum to the bilateral IFG, encompassing Broca's area on the left and its right-sided homologue. There were no correlations found between these pathways and phonological scores, important for reading ability, in either PT or TC. However, the strength of connectivity from the left cerebellar ROI to the bilateral IFG positively correlated with receptive language scores in PT (not in TC).

While not studies of connectivity metrics explicitly, some task-based fMRI studies have investigated differences in representation of the “reading network” in PT children vs. TC. Because definition of ROIs or “nodes” of the network of interest is a critical step in any functional connectivity analysis, the studies are briefly summarized. In a small study of adolescent male VPT compared to TC undergoing alternating visual phonologic processing tasks in fMRI (93), VPT demonstrated reduced activation in the left peristriate cortex, left cerebellum, and right precuneus with increased activation in the right hemisphere, precentral gyrus, and superior frontal cortex, whereas TC demonstrated greater activation in the peristriate cortex which includes the putative “visual word form area” which is important for reading in TC as demonstrated by decreased activation in children with dyslexia (39, 94).

In a study from a subset of the IVH Prevention cohort, Ment performed task-based fMRI in PT and TC at age 12 and correlated reading-related BOLD activation with behavioral metrics including silent reading, sight reading, decoding efficiency, phonological awareness and language scores (95). Compared to TC, VPT demonstrated reduced activation in the left middle temporal gyrus, left angular gyrus, and posterior cingulate gyrus and reduced deactivation in the left inferior parietal lobule and right inferior frontal gyrus during semantic processing. During phonologic processing, VPT demonstrated alternative patterns of activation in the left middle and superior temporal gyri, right anterior middle temporal gyrus, and left parahippocampal gyrus whereas TC demonstrated widespread frontal and occipital deactivation.

Finally, a task-based fMRI study of Swedish PT and TC adolescents related activation to measures of reading accuracy and reaction time in addition to metrics of visuospatial processing and verbal comprehension related to reading ability (96). PT exhibited increased activation in the left IFG during phonologic processing, decreased activation in the right supramarginal gyrus for orthographic processing, and decreased activation in a different region of the left IFG during the semantic condition. For PT, higher semantic task accuracy was related to increased activation in the left angular gyrus. PT exhibited lower visuo-spatial scores vs. TC which were correlated with increased right supramarginal gyrus activation during the phonologic processing task. However, there was no difference between groups in accuracy or reading time during the task, suggesting that the atypical activation and deactivation demonstrated by PT may be compensatory.

### 3.3. Magnetoencephalography

This review identified one study evaluating connectivity using magnetoencephalography (MEG) which is included in Table 2. Using a previously discussed cohort (84), Frye evaluated PT and TC adolescents categorized both by reading ability and birth group (97). PT were divided into low-risk and high-risk based on neonatal complications. Participants performed real-word and non-word rhyme tasks during MEG with ROI analysis involving Broca's and prefrontal areas, frontal and supplementary motor areas, middle temporal gyrus, superior temporal and Heschl's gyri, in addition to supramarginal and angular gyri. The MEG metric assessed was number of dipoles (NOD). Among good and average readers, those born high-risk preterm had greater NOD, especially during the 250–350 ms latency, in the prefrontal area during the real word rhyme task than those born low-risk PT and TC. Similarly, among good and average readers during the non-word rhyme task, the high-risk PT group demonstrated greater NOD, particularly during the 350–450 ms latency, in the left prefrontal area the low-risk PT and TC. Poor readers across birth groups demonstrated lower NOD in the Broca's and left prefrontal areas and higher NOD in homologous right sided cortical regions. The authors suggest their findings may reflect compensatory mechanisms of frontal overactivation and reduced left lateralization. While the authors analyze time courses of electromagnetic brain activity, number of dipoles is not a commonly used MEG connectivity metric and might not be interpretable as a functional connectivity metric at all. There are no other studies evaluating connectivity on MEG related to reading in preterm children to which we can compare results.

## 4. Discussion

As neuroimaging techniques advance, dMRI, fMRI, and MEG have all been used to evaluate brain structure and function related to emergent literacy and reading measures in PT children who are known to be at risk for reading difficulty. Elucidation of the reading network in PT would help define typical development in this population and the neuroimaging correlates of such. Subsequently, biomarkers indicative of children at risk for poor reading may be identified, allowing earlier intervention and may potentially guiding effectiveness of future interventions. Though innovative work has been done to elucidate the reading network in PT and the differences from TC, much important work remains to fully characterize this process.

### 4.1. Structural connectivity and reading in prematurity

#### 4.1.1. Early literacy phase (6–8 years)

In studies of younger children, FA of the classical dorsal and ventral pathways positively associated with reading in TC are largely not associated with reading outcome in PT (57, 77, 79). FA of diffuse and widespread tracts were associated with reading skill in PT in one larger study, but correlations were not found using tensor-free metrics (81). One study was done evaluating relaxometry, a myelin-water fraction related metric, which demonstrated associations with reading in PT only, but associations were not found with FA alone (57).

#### 4.1.2. Conventional literacy phase (9 years and above)

Studies in older children and adolescents have focused on specific ROI. Several studies have associated increased FA of certain segments of the corpus callosum with better reading-related outcomes in PT (78, 86, 90). Interestingly, some studies in TC have demonstrated that good readers have lower FA in the corpus callosum than poor readers, which may represent pruning or decreased interhemispheric connections as reading ability matures (42, 43). Additionally, studies of language in PT have identified extra-callosal pathways which may serve as compensatory mechanisms for language function, possibly due to perinatal injury to the corpus callosum (49, 52). The cerebellum has been implicated in reading development in TC, with several studies implicating cerebellar structures as important in reading and emergent literacy skills (40, 41), and thus, has been explored in studies of PT. There are variable findings relating cerebellar structures with reading skill in PT, with one study showing positive associations of FA of the left ICP in PT and not TC (92) and another showing negative association with the left ICP and reading in both PT and TC (88). The findings are inconsistent. As cerebellar structures are associated with the contralateral cerebrum, we would have expected the right cerebellum to be associated with left lateralized language and reading pathways in older TC if not in PT. Of particular interest in many studies of PT are the classical dorsal and ventral tracts associated with reading outcome in TC. In general, negative associations were found between FA of these pathways (left sided SLF, AF, bilateral UF, bilateral CST) and reading outcome in TC while positive associations are seen with the same tracts and reading outcome in PT (82, 86, 87, 89). One explanation of the negative correlations with reading outcome might be that, in TC, pruning leads to decreased FA in important areas as efficiency increases, such that initial positive associations between FA and ability become negative associations in later years. Alternately, the network in TC could become more complex with increased crossing fibers resulting in lower FA. Notably, some of these studies demonstrate positive associations in PT adolescents between FA of right sided tracts and reading outcomes at an age when the tracts, specifically the SLF, have typically have left lateralized in TC, which may suggest delayed maturation (82, 89). However, other studies did not find such correlations with reading outcome (98). Reduced left lateralization of white matter tracts in children born PT compared to TC is among the most widely described findings in structural connectivity studies of older children and adolescents (85, 86, 97), consistent with literature in preterm children revealing reduced left lateralization of language-related pathways (45, 99). In TC with dyslexia, reduced lateralization of reading-related pathways has been demonstrated compared to typically developing peers, with increased bi-hemispheric involvement thought to represent strain or increased effort required read (39, 94, 100). Research reviewed above using higher-order tensor-free analysis of diffusion data did not find significant results in PT.

It should be noted that the reviewed studies are not truly longitudinal so the following comparisons between studies are speculative at best. It is possible seemingly contradictory findings in younger and older cohorts of PT represent a delay in typical development of reading-related pathways. In the early literacy phase, reading outcome appears to be positively correlated with FA in traditional white matter tracts of the reading network in TC, but no association is seen in PT. This seems to shift in later childhood and adolescence during the conventional literacy phase when reading fluency has been achieved, with findings of no or negative associations of FA of dorsal and ventral stream tracts with reading outcome in TC but positive associations of the same tracts with reading outcome in PT. Aside from a delay in typical development, another possibility is that PT harness alternative pathways that are not identified in studies that use *a priori* defined tracts or are not adequately identified by FA as a metric. It is possible as well that varied outcomes can be attributed to methodological differences between studies.

### 4.2. Functional connectivity and reading in prematurity

#### 4.2.1. Early literacy phase (6–8 years)

Functional MRI studies of reading in PT children involve relatively small sample sizes, typically of older children. We found only one study of functional connectivity in the early literacy phase. In this study, areas canonically related to language, such as Wernicke's area, had increased connectivity to right sided homologues and frontal areas in PT (44, 47). These areas of alternative functional connectivity were not successfully related to behavioral measures of reading or language.

#### 4.2.2. Conventional literacy phase (9 years and above)

Notably, the task-based functional connectivity study available in older children (47) is based on *a priori*-selected ROIs from the study in younger children (44). The alternative connectivity identified from Wernicke's area to the right SMG has been associated with language outcomes in PT, which are foundational skills that contribute to reading ability. Interestingly, in this study increased connectivity in the alternative pathway was negatively associated with language scores and degree of maternal education. This suggests that alternative connectivity pathways may be most heavily relied on by those PT with the greatest deficits.

In general, the supplemental task-based fMRI studies reviewed demonstrate diffuse and bilateral patterns of activation in TC relative to PT children. Indeed, PT children appear to activate areas different from TC during task-based imaging, with some studies identifying more frontal activation (95, 96) and increased activation in atypical areas (96, 101) which may represent an attempt to compensate for difficulty with phonologic tasks. The differences in task-based activation during reading-related tasks may guide future connectivity studies as nodes or ROIs for analysis.

A resting state ROI-based fMRI study revealed increased connectivity in PT between the left cerebellum and the bilateral IFG (Broca's area and the right sided homologue). Increased connectivity to frontal regions and activation in frontal regions has been theorized to represent increased strain during performance of difficult tasks (102). Phonologic processing is a crucial foundational skill for reading and an area in which PT commonly exhibit deficits (26, 27, 29). PT may overcome deficits in areas such as phonological processing with increased effort requiring recruitment of bilateral and diffuse regions or alternative pathways compared to TC, which may explain the patterns seen in these studies. Interestingly, though, the left cerebellum was implicated in PT, in accordance with a structural connectivity study implicating left cerebellum as related to reading in younger children (79). As language and reading functions are typically left lateralized in TC, the increased connectivity related to the left cerebellum in PT is an unexpected finding that may speak to a more diffuse and less lateralized reading network in PT.

While not as commonly used as fMRI, MEG is a powerful tool to assess function supporting reading in PT. We identified only one study that used MEG in this review, yet the outcome measure of number of dipoles and settings used render interpretation difficult (97). The finding of greater NOD in prefrontal regions in high-risk preterm children who are good and average readers is thought to indicate increased frontal control required in good readers who are at risk. Likewise, the findings in poor readers in the high-risk preterm group of lower NOD in canonical left sided language regions and higher NOD in right-sided homologues is suggested to represent decreased lateralization and possibly increased strain related to reading.

### 4.3. Fundamental issues and problems

The literature surrounding connectivity related to reading outcomes in PT children has several shortcomings and remains limited, particularly as related to functional connectivity. One issue involves study design, as most of these studies are not longitudinal; thus, direct comparisons between studies cannot be made. The findings discussed represent a snapshot in time and it is difficult to determine if the differences seen between PT and TC represent a delay in maturation, alternate development, or simply variation between methodologies. Among diffusion studies, there is one longitudinal study with imaging at 6 and 8 years with consistent association patterns on dMRI, though not compared directly, but the relaxometry data with findings in PT was not followed serially (57). Thus, there are limited studies which might answer the question of whether the findings reported change over time as children develop and advance in their reading ability. Likewise, the functional imaging studies involve various subsets of a longitudinal cohort but varying tasks and populations prevent longitudinal analysis.

There is considerable heterogeneity in terms of population, neuroimaging method, and behavioral metrics used to evaluate reading ability. Several of these studies involve cohorts of children with wide age ranges from 9 to 17 years (86, 87, 89, 90). Some argue reading attainment occurs in 3 phrases—reading acquisition from age 3–6, reading refinement from age 6–14, and reading maintenance from age 14–21 (38). Thus, these broad cohorts may include children at various stages of reading development and the tract profiles could vary based on age. Additionally, cohorts studied include wide ranges of gestational age in the PT group, with several studies including any child less than 36 weeks, though late preterm infants are far less likely to have cognitive issues than VPT or especially EPT (86, 87, 89, 90). There are no studies investigating findings in EPT specifically, who are the most prone to reading difficulty (21, 26, 103). Finally, most of the studies employ small sample sizes which limits the significance of the results obtained.

Aside from small sample sizes and population heterogeneity, there are relatively few cohorts evaluated. Most of the available studies regarding reading-related connectivity in PT compared to TC stem from 3 cohorts—2 different cohorts analyzed at Stanford, including an older cohort from a study of long-term cognitive outcomes of prematurity (86–90) and a newer cohort to investigate the neural basis of reading (57, 77, 78, 79), and the follow up cohort of the Multicenter Indomethacin IVH Prevention trial (44, 47, 85, 92, 95, 101). Of the 22 studies evaluated in this review, 14 stem from one of these 3 cohorts, as do all the case reports mentioned. The remaining 8 studies include 3 from a single Australian cohort (80, 81, 91), 2 from a Houston cohort (84, 97), and 3 individual studies unrelated to other cohorts (82, 93, 96). It is possible that the results obtained thus far will not generalize given the relatively limited number of children studied.

Additionally, the studies reported use a wide range of metrics to assess reading. There are myriad tools that can be used to assess a variety of reading-related metrics, including sight reading, rapid naming, decoding, phonological awareness, and reading efficiency, fluency, and comprehension in both oral and silent reading. It is difficult to completely parse out the skills being assessed in many of the studies, especially as reading ability also encompasses language skills (vocabulary and verbal comprehension) in addition to executive function related skills such as attention.

Related to structural connectivity, most of the diffusion imaging studies use FA as the primary metric. FA is difficult to interpret when there are crossing fibers or branching axons as the averaged direction of diffusion in the voxel may not fully reflect the underlying tracts. In particular, analysis with measures other than FA are needed to assess the corpus callosum, as interhemispheric pathways containing interdigitated fibers are not be well assessed by this metric. Similarly, large axonal diameters may have increased perpendicular diffusion and may not be reflected by increasing FA. The studies involving DTI acknowledge these potential pitfalls, as DTI makes inferences about white matter integrity as a proxy for strength of connections but does not measure quantitative connectivity.

To combat this, some studies have employed advanced diffusion metrics to address the issues with using FA. Two studies used NODDI, which is theorized to account for axon density and dispersion that may cause FA to be unreliable (91). Another study used relaxometry, or measurement of the inverse of the time constant (R1), which is a myelin water fraction related metric, and found different results between tracts in PT and TC using FA and using R1 (57). In theory, myelin water fraction imaging should most accurately reflect underlying myelin content of tracts based on histologic measurements (56, 104). Myelin water fraction imaging has not been extensively used to evaluate myelin content as related to reading skill in PT but has been used to investigate differences between term typical readers and children with dyslexia (59). Higher-order, tensor-free techniques for the analysis of diffusion MRI data may also provide improved characterization of underlying myelin content by providing a quantitative measure of diffusion that is sensitive to crossing fibers and has been used in some studies assessing language in PT (49). Advanced diffusion imaging represents a burgeoning area of study which may provide more reliable metrics to assess white matter integrity and structural connectivity in future studies. However, diffusion imaging has limitations despite improving metrics and in some cases, functional imaging may be a more ideal method to analyze processes that require rapid integration of information from multiple areas of the brain such as reading.

Task-based fMRI studies can assess the areas of the brain that activate during specific reading tasks in order to better characterize functional connectivity, instead of surmising which tracts may be important based on data obtained from TC (or even term-born adults) about the reading network. A downfall of the existing studies is the reliance of connectivity analysis based on pre-selected ROIs, which limits the analysis to areas known to be involved in reading based on prior studies and may miss atypical pathways harnessed by PT outside the ROIs. Resting state fMRI connectivity studies may also provide important information about intrinsic connectivity. However, the number of studies available are quite limited. There are also no studies evaluating functional connectivity metrics in cohorts of PT children in the emergent literary phase reading development and a single study in the early literacy phase. There are extremely limited studies using MEG to evaluate reading-related connectivity in PT children of any age.

### 4.4. Research gaps

In terms of study design, development of the reading pathway in preterm children would ideally be evaluated by serial imaging and cognitive assessments throughout the development of reading ability, beginning pre-literacy and following into the reading maintenance stage. Unfortunately, such longitudinal cohort studies with serial testing of a population over time are difficult to accomplish and do not currently exist. Our review identified a series of fMRI studies performed at different ages but in various subsets of the follow up cohort of the Multicenter Indomethacin IVH Prevention Trial, so longitudinal analysis was not possible. There are a series of articles on 2 different cohorts analyzed at Stanford, one aged 9–17 and one cohort with scans at age 6 and repeat testing at age 8, but to date there has not been longitudinal, serial imaging in these groups. Though time intensive and challenging, longitudinal studies are needed to better define development of the reading network in PT children and allow direct comparison regarding whether the differences seen in connectivity vs. TC are due to delayed maturation or alternative development.

Additionally, there are no studies restricting to EPT children. Based on our knowledge of cognitive outcomes, these children are at highest risk for poor reading and should be an area of focus. Most studies assess high functioning children and often exclude those with significant brain injuries. While this approach provides important information about a relatively pure effect of prematurity on development, there is likely much to be learned about compensatory mechanisms in children with significant IVH, ventriculomegaly, or PVL, especially in those who achieve a normal or near normal cognitive outcome. Several case reports from children excluded from cohort studies for high grade IVH, ventriculomegaly, or missing pathways reveal interesting findings that may indicate compensatory pathways (105–107). One case report discusses a child with an absent SLF and AF bilaterally, dorsal pathways thought to be crucial for phonologic processing and verbal repetition, who achieved average scores on language and reading testing with therapy (108). Reports of preterm children who suffered brain injury have the power to enhance our knowledge of development and suggest compensatory pathways. Ultimately, PT with history of brain injury are at highest risk for developmental delay in areas such as reading and may benefit the most from early identification and interventions. It is important to extend studies to this population in large enough sample sizes to draw conclusions.

Finally, identification of the ideal method to evaluate connectivity in PT remains elusive. DTI, while widely available, may not be the most accurate method of characterizing the underlying white matter tracts. Advanced diffusion imaging, such as NODDI, and myelin water fraction imaging may also provide better estimations of myelin content and white matter integrity, allowing improved assessment of white matter microstructure and structural connectivity especially in combination with other methods. More functional connectivity studies are needed. Regarding functional imaging, fMRI has excellent spatial resolution and the benefit of being able to track activation data during tasks, but suboptimal temporal resolution. MEG has excellent temporal resolution though poorer spatial resolution and is not as widely available or as commonly used. No studies exist that combine structural and functional connectivity analysis. Multimodal studies that combine various neuroimaging methods may help to further elucidate how findings vary between groups.

### 4.5. Future directions

Advanced, tensor-free analytical models may overcome some of the existing issues with DTI. Combination of these approaches may lead to improved understanding of the structural connectome that underlies reading in PT. Studies that use multiple modalities, such as diffusion imaging combined with functional imaging with fMRI or MEG, may help further clarify the variability in structural and functional connectivity seen in PT and TC. MEG has not been widely used to date to evaluate reading-related connectivity in PT, though there are a series of studies using fMRI-constrained MEG to assess functional connectivity of language in this population (48, 50, 51). These studies use a data-driven approach to connectivity analysis by harnessing areas of fMRI activation during language tasks as nodes to guide the connectivity analysis in MEG. This approach capitalizes on the relative strengths of each modality by combining the excellent spatial resolution of fMRI with the unmatched temporal resolution of MEG. This model may be ideally suited to evaluate related networks, such as reading, in which alternative connectivity is suspected as the analysis is not limited by *a priori* defined networks, and could be combined with assessments of structural connectivity.

As we have noted above, in order to assess the development of reading networks over time, longitudinal studies of larger cohorts are needed that perform serial imaging in the same children. Such studies should include at-risk populations such as ELBW or EPT children and PT with brain injury who have often been excluded. Ideally, imaging would begin in the pre-literacy phase, or even in the immediate postnatal period, and be obtained serially with cognitive assessments to track development of the reading network. Recent studies in PT suggest that white matter differences at term-corrected age in the AF and ILF are related to language outcome at age 2 (109, 110). Continued research may ultimately be used to guide early intervention. This could be extended to reading networks and pathways as children develop these skills.

The existing literature surrounding reading disability and difficulty in preterm children acknowledge the coexistence of reading difficulty with other deficits such as in executive function, attention, or other specific learning disabilities, such as mathematics (13, 18). Though we focused in this review on outcomes specifically related to reading, we acknowledge that reading ability is related to other skills and these relationships will be important to tease out in future studies. Executive function skills may modulate reading ability (111). Further characterization of the networks and skillsets crucial to reading in PT may help lead to interventions that optimize reading ability. For example, phonological awareness is a skill that is crucial to future reading (27, 29). An exploratory study involving working memory training in PT preschoolers resulted in a short-term improvement in executive function and phonological awareness (112). It is possible that reading outcome may be improved by interventions both for skills classically associated with reading, such as phonological awareness, and for related skills such as executive function, attention, and working memory. In addition, important information about the home environment, language exposure, and cognitive stimulation should be obtained to assess the role of the environment in development of reading in PT, especially as some have emphasized the importance of maternal education, which can be seen as a marker of home cognitive stimulation (47). The home environment, including early language exposure, cognitive stimulation, and digital media use, has been shown to be critical in the development of reading in TC and may to be an important factor for PT as well (62, 113, 114).

## 5. Conclusion

As survival of premature infants has improved, focus has shifted to neurodevelopmental impairments and interventions which may improve outcomes. Reading and literacy are crucial skills for academic achievement and social functioning, with poor reading associated with many adverse outcomes (62). Given PT are at increased risk for language and reading difficulty (26), neuroimaging using TC as a comparison may help identify the underlying mechanisms of the persistent reading delays exhibited in PT. Studies of structural and functional brain connectivity related to reading outcomes in PT have revealed differences in reading-related pathways, which may represent delayed maturation of typical pathways or the development of alternative mechanisms for reading. Longitudinal studies are needed using advanced diffusion imaging, fMRI, and MEG to better characterize the connectome underlying reading in PT and identify compensatory mechanisms. The goal of these future longitudinal studies involving multimodal imaging would be to identify the components of the reading network in PT, to elucidate biomarkers of resiliency that can be recognized before children have attained reading proficiency, and – perhaps most importantly—to identify modifiable factors that can be targeted by interventions to improve reading outcome in this vulnerable population.

## References

[1] Adams-Chapman I Heyne RJ DeMauro SB Duncan AF Hintz SR Pappas A Neurodevelopmental impairment among extremely preterm infants in the neonatal research network. Pediatrics. (2018) 141(5):e20173091. 10.1542/peds.2017-3091 29666163 PMC5914487

[2] Pierrat V Marchand-Martin L Arnaud C Kaminski M Resche-Rigon M Lebeaux C Neurodevelopmental outcome at 2 years for preterm children born at 22 to 34 weeks’ gestation in France in 2011: EPIPAGE-2 cohort study. Br Med J. (2017) 358:j3448. 10.1136/bmj.j3448 28814566 PMC5558213

[3] Mercier CE Dunn MS Ferrelli KR Howard DB Soll RF, Group VONEIF-US. Neurodevelopmental outcome of extremely low birth weight infants from the Vermont Oxford network: 1998–2003. Neonatology. (2010) 97(4):329–38. 10.1159/000260136 19940516 PMC2889257

[4] Fleiss B Gressens P Stolp HB. Cortical gray matter injury in encephalopathy of prematurity: link to neurodevelopmental disorders. Front Neurol. (2020) 11:575. 10.3389/fneur.2020.00575 32765390 PMC7381224

[5] Volpe JJ. The encephalopathy of prematurity–brain injury and impaired brain development inextricably intertwined. Semin Pediatr Neurol. (2009) 16(4):167–78. 10.1016/j.spen.2009.09.005 19945651 PMC2799246

[6] Baburamani AA Supramaniam VG Hagberg H Mallard C. Microglia toxicity in preterm brain injury. Reprod Toxicol. (2014) 48:106–12. 10.1016/j.reprotox.2014.04.002 24768662 PMC4155935

[7] Vohr B. Speech and language outcomes of very preterm infants. Semin Fetal Neonatal Med. (2014) 19(2):78–83. 10.1016/j.siny.2013.10.007 24275068

[8] Joseph RM O’Shea TM Allred EN Heeren T Hirtz D Jara H Neurocognitive and academic outcomes at age 10 years of extremely preterm newborns. Pediatrics. (2016) 137(4):e20154343. 10.1542/peds.2015-4343 27006473 PMC4811321

[9] Luu TM Vohr BR Schneider KC Katz KH Tucker R Allan WC Trajectories of receptive language development from 3 to 12 years of age for very preterm children. Pediatrics. (2009) 124(1):333–41. 10.1542/peds.2008-2587 19564317 PMC2704989

[10] Pritchard VE Bora S Austin NC Levin KJ Woodward LJ. Identifying very preterm children at educational risk using a school readiness framework. Pediatrics. (2014) 134(3):e825–32. 10.1542/peds.2013-3865 25113296

[11] Garfield CF Karbownik K Murthy K Falciglia G Guryan J Figlio DN Educational performance of children born prematurely. JAMA Pediatr. (2017) 171(8):764–70. 10.1001/jamapediatrics.2017.1020 28604933 PMC5710633

[12] Mallinson DC Grodsky E Ehrenthal DB. Gestational age, kindergarten-level literacy, and effect modification by maternal socio-economic and demographic factors. Paediatr Perinat Epidemiol. (2019) 33(6):467–79. 10.1111/ppe.12588 31503367 PMC6823120

[13] Aarnoudse-Moens CS Weisglas-Kuperus N van Goudoever JB Oosterlaan J. Meta-analysis of neurobehavioral outcomes in very preterm and/or very low birth weight children. Pediatrics. (2009) 124(2):717–28. 10.1542/peds.2008-2816 19651588

[14] Anderson P Doyle LW Group VICS. Neurobehavioral outcomes of school-age children born extremely low birth weight or very preterm in the 1990s. JAMA. (2003) 289(24):3264–72. 10.1001/jama.289.24.3264 12824207

[15] Saigal S den Ouden L Wolke D Hoult L Paneth N Streiner DL School-age outcomes in children who were extremely low birth weight from four international population-based cohorts. Pediatrics. (2003) 112(4):943–50. 10.1542/peds.112.4.943 14523190

[16] Johnson S Strauss V Gilmore C Jaekel J Marlow N Wolke D. Learning disabilities among extremely preterm children without neurosensory impairment: comorbidity, neuropsychological profiles and scholastic outcomes. Early Hum Dev. (2016) 103:69–75. 10.1016/j.earlhumdev.2016.07.009 27517525

[17] Johnson S Hennessy E Smith R Trikic R Wolke D Marlow N. Academic attainment and special educational needs in extremely preterm children at 11 years of age: the EPICure study. Arch Dis Child Fetal Neonatal Ed. (2009) 94(4):F283–9. 10.1136/adc.2008.152793 19282336

[18] Akshoomoff N Joseph RM Taylor HG Allred EN Heeren T O'Shea TM Academic achievement deficits and their neuropsychological correlates in children born extremely preterm. J Dev Behav Pediatr. (2017) 38(8):627–37. 10.1097/DBP.0000000000000479 28877090 PMC5646684

[19] Carter FA Msall ME. Long-Term functioning and participation across the life course for preterm neonatal intensive care unit graduates. Clin Perinatol. (2018) 45(3):501–27. 10.1016/j.clp.2018.05.009 30144852 PMC11160115

[20] Aarnoudse-Moens CS Oosterlaan J Duivenvoorden HJ van Goudoever JB Weisglas-Kuperus N. Development of preschool and academic skills in children born very preterm. J Pediatr. (2011) 158(1):51–6. 10.1016/j.jpeds.2010.06.052 20708749

[21] Aylward GP. Cognitive and neuropsychological outcomes: more than IQ scores. Ment Retard Dev Disabil Res Rev. (2002) 8(4):234–40. 10.1002/mrdd.10043 12454899

[22] Pritchard VE Clark CA Liberty K Champion PR Wilson K Woodward LJ. Early school-based learning difficulties in children born very preterm. Early Hum Dev. (2009) 85(4):215–24. 10.1016/j.earlhumdev.2008.10.004 19022593

[23] Luu TM Ment LR Schneider KC Katz KH Allan WC Vohr BR. Lasting effects of preterm birth and neonatal brain hemorrhage at 12 years of age. Pediatrics. (2009) 123(3):1037–44. 10.1542/peds.2008-1162 19255037 PMC2651566

[24] Vohr BR Allan WC Westerveld M Schneider KC Katz KH Makuch RW School-age outcomes of very low birth weight infants in the indomethacin intraventricular hemorrhage prevention trial. Pediatrics. (2003) 111(4 Pt 1):e340–6. 10.1542/peds.111.4.e340 12671149

[25] Litt J Taylor HG Klein N Hack M. Learning disabilities in children with very low birthweight: prevalence, neuropsychological correlates, and educational interventions. J Learn Disabil. (2005) 38(2):130–41. 10.1177/00222194050380020301 15813595

[26] Kovachy VN Adams JN Tamaresis JS Feldman HM. Reading Abilities in school-aged preterm children: a review and meta-analysis. Dev Med Child Neurol. (2015) 57(5):410–9. 10.1111/dmcn.12652 25516105 PMC4397135

[27] Wocadlo C Rieger I. Phonology, rapid naming and academic achievement in very preterm children at eight years of age. Early Hum Dev. (2007) 83(6):367–77. 10.1016/j.earlhumdev.2006.08.001 16979856

[28] Borchers LR Bruckert L Travis KE Dodson CK Loe IM Marchman VA Predicting text reading skills at age 8 years in children born preterm and at term. Early Hum Dev. (2019) 130:80–6. 10.1016/j.earlhumdev.2019.01.012 30708270 PMC6402954

[29] Guarini A Sansavini A Fabbri C Savini S Alessandroni R Faldella G Long-term effects of preterm birth on language and literacy at eight years. J Child Lang. (2010) 37(4):865–85. 10.1017/S0305000909990109 19698208

[30] Whitehurst GJ Lonigan CJ. Child development and emergent literacy. Child Dev. (1998) 69(3):848–72. 10.1111/j.1467-8624.1998.tb06247.x 9680688

[31] McKean C Reilly S Bavin EL Bretherton L Cini E Conway L Language outcomes at 7 years: early predictors and co-occurring difficulties. Pediatrics. (2017) 139(3):e20161684. 10.1542/peds.2016-1684 28179482

[32] Joensuu E Munck P Setänen S Lipsanen J Huhtala M Lapinleimu H Associations between language at 2 years and literacy skills at 7 years in preterm children born at very early gestational age and/or with very low birth weight. Children (Basel). (2021) 8(6):510. doi: 10.3390/children8060510 34208622 PMC8233950

[33] Eadie P Bavin EL Bretherton L Cook F Gold L Mensah F Predictors in infancy for language and academic outcomes at 11 years. Pediatrics. (2021) 147(2):e20201712. 10.1542/peds.2020-1712 33431588

[34] Safi D Lefebvre P Nader M. Literacy acquisition: Reading development. Handb Clin Neurol. (2020) 173:185–99. 10.1016/B978-0-444-64150-2.00017-4 32958173

[35] Hickok G Poeppel D. Dorsal and ventral streams: a framework for understanding aspects of the functional anatomy of language. Cognition. (2004) 92(1–2):67–99. 10.1016/j.cognition.2003.10.011 15037127

[36] Horowitz-Kraus T Hutton JS. From emergent literacy to reading: how learning to read changes a child’s brain. Acta Paediatr. (2015) 104(7):648–56. 10.1111/apa.13018 25847632

[37] Vandermosten M Boets B Wouters J Ghesquière P. A qualitative and quantitative review of diffusion tensor imaging studies in reading and dyslexia. Neurosci Biobehav Rev. (2012) 36(6):1532–52. 10.1016/j.neubiorev.2012.04.002 22516793

[38] Cheema K Cummine J, for the Pediatric Imaging N, and Genetics Study. The relationship between white matter and Reading acquisition, refinement and maintenance. Dev Neurosci. (2018) 40(3):209–22. 10.1159/000489491 29940596

[39] Dehaene S. Inside the letterbox: how literacy transforms the human brain. Cerebrum. (2013) 2013:7. PMID: ; PMCID: 23847714 PMC3704307

[40] Hutton JS Phelan K Horowitz-Kraus T Dudley J Altaye M DeWitt T Story time turbocharger? Child engagement during shared reading and cerebellar activation and connectivity in preschool-age children listening to stories. PLoS One. (2017) 12(5):e0177398. 10.1371/journal.pone.0177398 28562619 PMC5451016

[41] Bruckert L Travis KE Mezer AA Ben-Shachar M Feldman HM. Associations of reading efficiency with white matter properties of the cerebellar peduncles in children. Cerebellum. (2020) 19(6):771–7. 10.1007/s12311-020-01162-2 32642932 PMC7606658

[42] Dougherty RF Ben-Shachar M Deutsch GK Hernandez A Fox GR Wandell BA. Temporal-callosal pathway diffusivity predicts phonological skills in children. Proc Natl Acad Sci USA. (2007) 104(20):8556–61. 10.1073/pnas.0608961104 17483487 PMC1895988

[43] Odegard TN Farris EA Ring J McColl R Black J. Brain connectivity in non-reading impaired children and children diagnosed with developmental dyslexia. Neuropsychologia. (2009) 47(8–9):1972–7. 10.1016/j.neuropsychologia.2009.03.009 19428430

[44] Gozzo Y Vohr B Lacadie C Hampson M Katz KH Maller-Kesselman J Alterations in neural connectivity in preterm children at school age. Neuroimage. (2009) 48(2):458–63. 10.1016/j.neuroimage.2009.06.046 19560547 PMC2775072

[45] Kwon SH Scheinost D Vohr B Lacadie C Schneider K Dai F Functional magnetic resonance connectivity studies in infants born preterm: suggestions of proximate and long-lasting changes in language organization. Dev Med Child Neurol. (2016) 58(Suppl 4):28–34. 10.1111/dmcn.13043 27027605 PMC6426123

[46] Vandormael C Schoenhals L Hüppi PS Filippa M Borradori Tolsa C. Language in preterm born children: atypical development and effects of early interventions on neuroplasticity. Neural Plast. (2019) 2019:6873270. 10.1155/2019/6873270 30930944 PMC6410465

[47] Myers EH Hampson M Vohr B Lacadie C Frost SJ Pugh KR Functional connectivity to a right hemisphere language center in prematurely born adolescents. Neuroimage. (2010) 51(4):1445–52. 10.1016/j.neuroimage.2010.03.049 20347043 PMC2872040

[48] Barnes-Davis ME Merhar SL Holland SK Kadis DS. Extremely preterm children exhibit increased interhemispheric connectivity for language: findings from fMRI-constrained MEG analysis. Dev Sci. (2018) 21(6):e12669. 10.1111/desc.12669 29659125 PMC6193851

[49] Barnes-Davis ME Williamson BJ Merhar SL Holland SK Kadis DS. Rewiring the extremely preterm brain: altered structural connectivity relates to language function. Neuroimage Clin. (2020) 25:102194. 10.1016/j.nicl.2020.102194 32032818 PMC7005506

[50] Barnes-Davis ME Merhar SL Holland SK Parikh NA Kadis DS. Extremely preterm children demonstrate hyperconnectivity during verb generation: a multimodal approach. Neuroimage Clin. (2021) 30:102589. 10.1016/j.nicl.2021.102589 33610096 PMC7903004

[51] Barnes-Davis ME Fujiwara H Drury G Merhar SL Parikh NA Kadis DS. Functional hyperconnectivity during a stories listening task in magnetoencephalography is associated with language gains for children born extremely preterm. Brain Sci. (2021) 11(10):1271. 10.3390/brainsci11101271 34679336 PMC8534020

[52] Barnes-Davis ME Williamson BJ Merhar SL Nagaraj UD Parikh NA Kadis DS. Extracallosal structural connectivity is positively associated with language performance in well-performing children born extremely preterm. Front Pediatr. (2022) 10:821121. 10.3389/fped.2022.821121 35372163 PMC8971711

[53] Feldman HM Yeatman JD Lee ES Barde LH Gaman-Bean S. Diffusion tensor imaging: a review for pediatric researchers and clinicians. J Dev Behav Pediatr. (2010) 31(4):346–56. 10.1097/DBP.0b013e3181dcaa8b 20453582 PMC4245082

[54] Jeurissen B Leemans A Tournier JD Jones DK Sijbers J. Investigating the prevalence of complex fiber configurations in white matter tissue with diffusion magnetic resonance imaging. Hum Brain Mapp. (2013) 34(11):2747–66. 10.1002/hbm.22099 22611035 PMC6870534

[55] Zhang H Schneider T Wheeler-Kingshott CA Alexander DC. NODDI: practical in vivo neurite orientation dispersion and density imaging of the human brain. Neuroimage. (2012) 61(4):1000–16. 10.1016/j.neuroimage.2012.03.072 22484410

[56] van der Weijden CWJ García DV Borra RJH Thurner P Meilof JF van Laar PJ Myelin quantification with MRI: a systematic review of accuracy and reproducibility. Neuroimage. (2021) 226:117561. 10.1016/j.neuroimage.2020.117561 33189927

[57] Brignoni-Pérez E Dubner SE Ben-Shachar M Berman S Mezer AA Feldman HM White matter properties underlying reading abilities differ in 8-year-old children born full term and preterm: a multi-modal approach. Neuroimage. (2022) 256:119240. 10.1016/j.neuroimage.2022.119240 35490913 PMC9213558

[58] Stüber C Morawski M Schäfer A Labadie C Wähnert M Leuze C Myelin and iron concentration in the human brain: a quantitative study of MRI contrast. Neuroimage. (2014) 93(Pt 1):95–106. 10.1016/j.neuroimage.2014.02.026 24607447

[59] Beaulieu C Yip E Low PB Mädler B Lebel CA Siegel L Myelin water imaging demonstrates lower brain myelination in children and adolescents with poor reading ability. Front Hum Neurosci. (2020) 14:568395. 10.3389/fnhum.2020.568395 33192398 PMC7596275

[60] Glover GH. Overview of functional magnetic resonance imaging. Neurosurg Clin N Am. (2011) 22(2):133–9, vii. 10.1016/j.nec.2010.11.001 21435566 PMC3073717

[61] Gaudet I Hüsser A Vannasing P Gallagher A. Functional brain connectivity of language functions in children revealed by EEG and MEG: a systematic review. Front Hum Neurosci. (2020) 14:62. 10.3389/fnhum.2020.00062 32226367 PMC7080982

[62] Hutton JS DeWitt T Hoffman L Horowitz-Kraus T Klass P. Development of an eco-biodevelopmental model of emergent literacy before kindergarten: a review. JAMA Pediatr. (2021) 175(7):730–41. 10.1001/jamapediatrics.2020.6709 33720328

[63] Hayward DV Stewart GE Phillips LM Norris SP Lovell MA. Test review: peabody picture vocabulary test-III (PPVT-III). Language, phonological awareness, and reading test directory. Edmonton, AB: Canadian Centre for Research on Literacy (2013).

[64] Coret MC McCrimmon AW. Test review: Wiig, E. H., Semel, E. & Secord, W. A. (2013). clinical evaluation of language fundamentals-fifth edition (CELF-5). J Psychoeduc Assess. (2015) 33(5):495–500. 10.1177/0734282914557616

[65] Tennant KE. Test review: comprehensive test of phonological processing–second edition (CTOPP-2). J Psychoeduc Assess. (2014) 32(7):678–91. 10.1177/0734282914525028

[66] Schuele CM Boudreau D. Phonological awareness intervention: beyond the basics. Lang Speech Hear Serv Sch. (2008) 39(1):3–20. 10.1044/0161-1461(2008/002) 18162644

[67] Hall AH Tannebaum RP. Test review: J. L. Wiederholt & B. R. Bryant. (2012). Gray oral reading tests—fifth edition (GORT-5). Austin, TX: pro-ed. J Psychoeduc Assess. (2013) 31(5):516–20. 10.1177/0734282912468578

[68] Abu-Hamour B Al Hmouz H Mattar J Muhaidat M. The use of Woodcock-Johnson tests for identifying students with special needs-a comprehensive literature review. Procedia Soc Behav Sci. (2012) 47:665–73. 10.1016/j.sbspro.2012.06.714

[69] Proger BB. Test review N o. 18 woodcock reading mastery test. J Spec Educ. (1975) 9(4):439–44.

[70] Tarar JM Meisinger EB Dickens RH. Test review: test of word reading efficiency-second edition (TOWRE-2) by Torgesen, J. K., Wagner, R. K., & Rashotte, C. A. J School Psychol. (2015) 30(4):320–6. 10.1177/0829573515594334

[71] Test review No. 4 peabody individual achievement test. J Spec Educ. (1970) 4(4):461–7. 10.1177/002246697000400412

[72] Dell CA Harrold B Dell T. Test review: Wilkinson, G. S. & Robertson, G. J. (2006). wide range achievement test-fourth edition. Lutz, FL: psychological assessment resources. WRAT4 introductory kit (includes manual, 25 test/response forms [blue and green], and accompanying test materials): $243. Rehabil Counsel Bull. (2008) 52(1):57–60. 10.1177/0034355208320076

[73] Allin M Matsumoto H Santhouse AM Nosarti C AlAsady MH Stewart AL Cognitive and motor function and the size of the cerebellum in adolescents born very pre-term. Brain. (2001) 124(Pt 1):60–6. 10.1093/brain/124.1.60 11133787

[74] Stewart AL Rifkin L Amess PN Kirkbride V Townsend JP Miller DH Brain structure and neurocognitive and behavioural function in adolescents who were born very preterm. Lancet. (1999) 353(9165):1653–7. 10.1016/S0140-6736(98)07130-X 10335784

[75] Kesler SR Vohr B Schneider KC Katz KH Makuch RW Reiss AL Increased temporal lobe gyrification in preterm children. Neuropsychologia. (2006) 44(3):445–53. 10.1016/j.neuropsychologia.2005.05.015 15985272

[76] Burger C Biermayr M Posod A Neubauer V Pupp Peglow U Kuenz K Amplitude-integrated electroencephalography shows alterations in children born preterm displaying poor literacy precursor skills. Acta Paediatr. (2019) 108(9):1661–8. 10.1111/apa.14755 30779217 PMC6767598

[77] Dodson CK Travis KE Borchers LR Marchman VA Ben-Shachar M Feldman HM. White matter properties associated with pre-reading skills in 6-year-old children born preterm and at term. Dev Med Child Neurol. (2018) 60(7):695–702. 10.1111/dmcn.13783 29722009 PMC5993607

[78] Dubner SE Dodson CK Marchman VA Ben-Shachar M Feldman HM Travis KE. White matter microstructure and cognitive outcomes in relation to neonatal inflammation in 6-year-old children born preterm. Neuroimage Clin. (2019) 23:101832. 10.1016/j.nicl.2019.101832 31075555 PMC6603335

[79] Bruckert L Borchers LR Dodson CK Marchman VA Travis KE Ben-Shachar M White matter plasticity in reading-related pathways differs in children born preterm and at term: a longitudinal analysis. Front Hum Neurosci. (2019) 13:139. 10.3389/fnhum.2019.00139 31139064 PMC6519445

[80] Thompson DK Loh WY Connelly A Cheong JLY Spittle AJ Chen J Basal ganglia and thalamic tract connectivity in very preterm and full-term children; associations with 7-year neurodevelopment. Pediatr Res. (2020) 87(1):48–56. 10.1038/s41390-019-0546-x 31486778

[81] Kelly CE Thompson DK Chen J Leemans A Adamson CL Inder TE Axon density and axon orientation dispersion in children born preterm. Hum Brain Mapp. (2016) 37(9):3080–102. 10.1002/hbm.23227 27133221 PMC5524572

[82] Kallankari H Taskila H-L Heikkinen M Hallman M Saunavaara V Kaukola T. Microstructural alterations in association tracts and language abilities in schoolchildren born very preterm and with poor fetal growth. Pediatr Radiol. (2022) 53(1):94–103. 10.1007/s00247-022-05418-3 35773359 PMC9816217

[83] Denckla MB Cutting LE. History and significance of rapid automatized naming. Ann. of Dyslexia. (1999):29–42. 10.1007/s11881-999-0018-9

[84] Frye RE Hasan K Malmberg B Desouza L Swank P Smith K Superior longitudinal fasciculus and cognitive dysfunction in adolescents born preterm and at term. Dev Med Child Neurol. (2010) 52(8):760–6. 10.1111/j.1469-8749.2010.03633.x 20187879 PMC2910222

[85] Mullen KM Vohr BR Katz KH Schneider KC Lacadie C Hampson M Preterm birth results in alterations in neural connectivity at age 16 years. Neuroimage. (2011) 54(4):2563–70. 10.1016/j.neuroimage.2010.11.019 21073965 PMC3020252

[86] Feldman HM Lee ES Yeatman JD Yeom KW. Language and reading skills in school-aged children and adolescents born preterm are associated with white matter properties on diffusion tensor imaging. Neuropsychologia. (2012) 50(14):3348–62. 10.1016/j.neuropsychologia.2012.10.014 23088817 PMC3631607

[87] Yeatman JD Dougherty RF Myall NJ Wandell BA Feldman HM. Tract profiles of white matter properties: automating fiber-tract quantification. PLoS One. (2012) 7(11):e49790. 10.1371/journal.pone.0049790 23166771 PMC3498174

[88] Travis KE Leitner Y Feldman HM Ben-Shachar M. Cerebellar white matter pathways are associated with reading skills in children and adolescents. Hum Brain Mapp. (2015) 36(4):1536–53. 10.1002/hbm.22721 25504986 PMC4374012

[89] Travis KE Ben-Shachar M Myall NJ Feldman HM. Variations in the neurobiology of reading in children and adolescents born full term and preterm. Neuroimage Clin. (2016) 11:555–65. 10.1016/j.nicl.2016.04.003 27158588 PMC4845391

[90] Andrews JS Ben-Shachar M Yeatman JD Flom LL Luna B Feldman HM. Reading Performance correlates with white-matter properties in preterm and term children. Dev Med Child Neurol. (2010) 52(6):e94–e100. 10.1111/j.1469-8749.2009.03456.x 19747208 PMC2892255

[91] Collins SE Spencer-Smith M Mürner-Lavanchy I Kelly CE Pyman P Pascoe L White matter microstructure correlates with mathematics but not word reading performance in 13-year-old children born very preterm and full-term. Neuroimage Clin. (2019) 24:101944. 10.1016/j.nicl.2019.101944 31426019 PMC6706654

[92] Constable RT Vohr BR Scheinost D Benjamin JR Fulbright RK Lacadie C A left cerebellar pathway mediates language in prematurely-born young adults. Neuroimage. (2013) 64:371–8. 10.1016/j.neuroimage.2012.09.008 22982585 PMC3508203

[93] Rushe TM Temple CM Rifkin L Woodruff PW Bullmore ET Stewart AL Lateralisation of language function in young adults born very preterm. Arch Dis Child Fetal Neonatal Ed. (2004) 89(2):F112–8. 10.1136/adc.2001.005314 14977893 PMC1756037

[94] Monzalvo K Fluss J Billard C Dehaene S Dehaene-Lambertz G. Cortical networks for vision and language in dyslexic and normal children of variable socio-economic status. Neuroimage. (2012) 61(1):258–74. 10.1016/j.neuroimage.2012.02.035 22387166

[95] Ment LR Peterson BS Vohr B Allan W Schneider KC Lacadie C Cortical recruitment patterns in children born prematurely compared with control subjects during a passive listening functional magnetic resonance imaging task. J Pediatr. (2006) 149(4):490–8. 10.1016/j.jpeds.2006.06.007 17011320 PMC2386989

[96] van Ettinger-Veenstra H Widén C Engström M Karlsson T Leijon I Nelson N. Neuroimaging of decoding and language comprehension in young very low birth weight (VLBW) adolescents: indications for compensatory mechanisms. PLoS One. (2017) 12(10):e0185571. 10.1371/journal.pone.0185571 28968426 PMC5624616

[97] Frye RE Malmberg B Desouza L Swank P Smith K Landry S. Increased prefrontal activation in adolescents born prematurely at high risk during a reading task. Brain Res. (2009) 1303:111–9. 10.1016/j.brainres.2009.09.091 19796631 PMC2783693

[98] Constable RT Ment LR Vohr BR Kesler SR Fulbright RK Lacadie C Prematurely born children demonstrate white matter microstructural differences at 12 years of age, relative to term control subjects: an investigation of group and gender effects. Pediatrics. (2008) 121(2):306–16. 10.1542/peds.2007-0414 18245422

[99] Rogers CE Lean RE Wheelock MD Smyser CD. Aberrant structural and functional connectivity and neurodevelopmental impairment in preterm children. J Neurodev Disord. (2018) 10(1):38. 10.1186/s11689-018-9253-x 30541449 PMC6291944

[100] Finn ES Shen X Holahan JM Scheinost D Lacadie C Papademetris X Disruption of functional networks in dyslexia: a whole-brain, data-driven analysis of connectivity. Biol Psychiatry. (2014) 76(5):397–404. 10.1016/j.biopsych.2013.08.031 24124929 PMC3984371

[101] Peterson BS Vohr B Kane MJ Whalen DH Schneider KC Katz KH A functional magnetic resonance imaging study of language processing and its cognitive correlates in prematurely born children. Pediatrics. (2002) 110(6):1153–62. 10.1542/peds.110.6.1153 12456913

[102] Miller EK Cohen JD. An integrative theory of prefrontal cortex function. Annu Rev Neurosci. (2001) 24(1):167–202. 10.1146/annurev.neuro.24.1.167 11283309

[103] Klebanov PK Brooks-Gunn J McCormick MC. School achievement and failure in very low birth weight children. J Dev Behav Pediatr. (1994) 15(4):248–56. 10.1097/00004703-199408000-00005 7798370

[104] MacKay AL Laule C. Magnetic resonance of myelin water: an. Brain Plast. (2016) 2(1):71–91. 10.3233/BPL-160033 29765849 PMC5928546

[105] Myall NJ Yeom KW Yeatman JD Gaman-Bean S Feldman HM. Case series: fractional anisotropy along the trajectory of selected white matter tracts in adolescents born preterm with ventricular dilation. J Child Neurol. (2013) 28(6):774–80. 10.1177/0883073812449693 22859695 PMC4277874

[106] Travis KE Leitner Y Ben-Shachar M Yeom KW Feldman HM. Case series: fractional anisotropy profiles of the cerebellar peduncles in adolescents born preterm with ventricular dilation. J Child Neurol. (2016) 31(3):321–7. 10.1177/0883073815592223 26116381 PMC4691425

[107] Travis KE Adams JN Ben-Shachar M Feldman HM. Decreased and increased anisotropy along major cerebral white matter tracts in preterm children and adolescents. PLoS One. (2015) 10(11):e0142860. 10.1371/journal.pone.0142860 26560745 PMC4641645

[108] Yeatman JD Feldman HM. Neural plasticity after pre-linguistic injury to the arcuate and superior longitudinal fasciculi. Cortex. (2013) 49(1):301–11. 10.1016/j.cortex.2011.08.006 21937035 PMC3259257

[109] Salvan P Tournier JD Batalle D Falconer S Chew A Kennea N Language ability in preterm children is associated with arcuate fasciculi microstructure at term. Hum Brain Mapp. (2017) 38(8):3836–47. 10.1002/hbm.23632 28470961 PMC5518442

[110] Dubner SE Rose J Bruckert L Feldman HM Travis KE. Neonatal white matter tract microstructure and 2-year language outcomes after preterm birth. Neuroimage Clin. (2020) 28:102446. 10.1016/j.nicl.2020.102446 33035964 PMC7554644

[111] Mürner-Lavanchy I Ritter BC Spencer-Smith MM Perrig WJ Schroth G Steinlin M Visuospatial working memory in very preterm and term born children–impact of age and performance. Dev Cogn Neurosci. (2014) 9:106–16. 10.1016/j.dcn.2014.02.004 24631800 PMC6989762

[112] Grunewaldt KH Løhaugen GC Austeng D Brubakk AM Skranes J. Working memory training improves cognitive function in VLBW preschoolers. Pediatrics. (2013) 131(3):e747–54. 10.1542/peds.2012-1965 23400616

[113] Hutton JS Dudley J Horowitz-Kraus T DeWitt T Holland SK. Associations between home literacy environment, brain white matter integrity and cognitive abilities in preschool-age children. Acta Paediatr. (2020) 109(7):1376–86. 10.1111/apa.15124 31854046 PMC7318131

[114] Vohr BR McGowan EC Bann C Das A Higgins R Hintz S Association of high screen-time use with school-age cognitive, executive function, and behavior outcomes in extremely preterm children. JAMA Pediatr. (2021) 175(10):1025–34. 10.1001/jamapediatrics.2021.2041 34251406 PMC8276120

